# Genomic diversity and evolutionary patterns of Edwardsiella ictaluri affecting farmed striped catfish (Pangasianodon hypophthalmus) in Vietnam over 20 years

**DOI:** 10.1099/mgen.0.001368

**Published:** 2025-02-19

**Authors:** Christopher J. Payne, Vo Hong Phuong, Nguyen Ngoc Phuoc, Tu Thanh Dung, Le Hong Phuoc, Margaret Crumlish

**Affiliations:** 1 Institute of Aquaculture, Faculty of Natural Sciences, University of Stirling, Stirling, UK; 2 Southern Monitoring Center for Aquaculture Environment and Epidemic, Research Institute for Aquaculture No. 2, Ho Chi Minh City, Vietnam; 3 Faculty of Fisheries, University of Agriculture and Forestry, Hue University, Hue, Vietnam; 4 Faculty of Aquatic Pathology, College of Aquaculture and Fisheries, Can Tho University, Can Tho, Vietnam

**Keywords:** antimicrobial resistance, *Edwardsiella ictaluri*, genome, *Pangasianodon hypophthalmus*, phylogenomics, virulence

## Abstract

*Edwardsiella ictaluri* continues to pose a significant risk to the health and production of striped catfish (*Pangasianodon hypophthalmus*) in Vietnam. Whilst recent advances in genomic sequencing provide an insight into the global genomic diversity of this important fish pathogen, genome-wide analysis of Vietnamese isolates recovered over time is lacking. In this study, we used a whole-genome sequencing approach to compare the genomes of 31 *E. ictaluri* isolates recovered over a 20-year period (2001–2021) and performed comparative genomic analysis to explore temporal changes in genome diversity, population structure and mechanisms driving pathogenesis and antimicrobial resistance. Our findings revealed an open pan-genome with 4148 genes and a core genome (3 060 genes) accounting for over two-thirds of the genome. Moreover, we found the genomes sequenced to classify into two distinct lineages and estimated the ancestral origin of these lineages within Vietnam to date back to the 1950s. Plasmids were highly prevalent in Vietnamese *E. ictaluri*, with isolates harbouring up to four plasmids within their genome. Further, a diverse mobilome was observed with nine different plasmid types detected across the genome collection. Exploration of putative plasmids revealed a diverse set of antimicrobial resistance genes (ARGs) against key antibiotics used in Vietnamese aquaculture and virulence genes associated with protein secretion systems. Correlation analysis revealed the total number of ARGs detected in genomes to increase with isolate recovery time. Whilst the number of virulence genes remained relatively stable, temporal variation was noted in several virulence factors related to motility and immune system modulation. Findings from this study highlight the need for continued genomic surveillance to monitor changes in antimicrobial resistance and pathogenesis, to help inform the development of disease control and management strategies.

**Impact Statement**

*Edwardsiella ictaluri* is an economically important fish pathogen and aetiological agent of Bacillary Necrosis of *Pangasius* (BNP), which continues to pose a serious threat to the production of striped catfish (*Pangasianodon hypophthalmus*) in Vietnam. Whilst BNP was first reported in 2001, genome-wide analysis of the genomic variation in *E. ictaluri* over time is currently lacking. In this study, we sequenced the complete genomes of 31 *E. ictaluri* recovered over a 20-year period (2001–2021). We then performed comparative genomic analysis, investigating the *E. ictaluri* pan-genome and temporal changes in the mobilome, resistome and virulome of isolates circulating in Vietnam over time. We showed that Vietnamese *E. ictaluri* has an open pan-genome, driven largely by a diverse mobilome. We also demonstrated that *E. ictaluri* isolates belonged to two distinct lineages, which could be traced back to the 1950s. We revealed that predicted antibiotic resistance in *E. ictaluri* has increased over time, as the number of antimicrobial resistance genes detected in genomes increased in isolates associated with more recent disease outbreaks. Whilst the number of virulence genes has remained relatively stable in Vietnamese *E. ictaluri* over two decades, temporal changes in virulence factors related to motility and immune modulation were detected. This study has considerably expanded the available genomic data of *E. ictaluri* in Vietnam, supporting genomic surveillance programmes to inform on changes from disease control and management strategies applied in the striped catfish sector.

## Data Summary

Whole-genome sequencing data generated in the study are available in the NCBI Sequence Read Archive repository under the accession number PRJNA1101888, and the accession numbers for all data sets used are provided in Tables 1 and S1, available in the online Supplementary Material.

**Table 1.** Genomic features of *Edwardsiella ictaluri* isolates recovered from *Pangasianodon hypophthalmus* in Vietnam (2001–2021)

Isolate	Year	Farm^*^	Province^†^	Completeness(%)	Contamination(%)	Size(Mbp)	Chromosome(Mbp)	Genomecoverage(X)	G+C %	CDSs(total)	rRNAgenes	tRNAgenes	Genomeaccession
Ei-104	2021	DT14	DT	98.5	1.2	3.93	3.75	1140	57.01	3623	25	95	CP152228-CP152231
Ei-103	2021	DT14	DT	98.4	1.1	3.96	3.78	899	57.00	3629	25	97	CP152232-CP152235
Ei-84	2020	AG9	AG	98.6	1.2	3.89	3.80	924	57.19	3551	25	97	CP152225-CP152227
Ei-80	2020	AG9	AG	98.6	1.3	3.91	3.83	911	57.16	3576	25	97	CP152222-CP152224
Ei-77cfu1pp	2020	AG8	AG	98.6	1.3	3.91	3.83	1059	57.16	3577	25	97	CP152219-CP152221
Ei-71	2020	CT3	CT	98.6	1.1	3.82	3.81	986	57.27	3473	25	97	CP152217-CP152218
Ei-69	2020	CT3	CT	98.3	1.1	3.77	3.77	1068	57.26	3424	25	97	CP152215-CP152216
Ei-85	2020	DT10	DT	98.6	1.2	3.89	3.80	934	57.19	3538	25	97	CP152212-CP152214
Ei-89cfu1pp	2020	DT11	DT	98.6	1.2	3.89	3.80	904	57.19	3552	25	97	CP152209-CP152211
Ei-90	2020	DT11	DT	98.6	1.2	3.89	3.80	1023	57.19	3553	25	97	CP152206-CP152208
Ei-96cfu2	2020	DT13	DT	98.4	1.1	3.84	3.75	874	57.19	3507	25	97	CP152203-CP152205
Ei-62	2020	VL7	VL	98.6	1.1	3.82	3.82	936	57.26	3474	28	98	CP152201-CP152202
Ei-64	2020	VL8	VL	98.6	1.1	4.05	3.81	1341	56.86	3739	25	95	CP152197-CP152200
Ei-60	2019	AG6	AG	98.6	1.0	3.88	3.72	1215	57.27	3575	25	97	CP152193-CP152196
Ei-59	2019	DT7	DT	98.6	1.0	3.88	3.72	1209	57.27	3576	25	97	CP152189-CP152192
Ei-57	2019	DT6	DT	98.6	1.2	3.91	3.82	719	57.16	3578	25	97	CP152186-CP152188
Ei-54	2018	TG4	TG	98.6	1.2	3.91	3.82	1126	57.16	3577	25	97	CP152183-CP152185
Ei-VN5	2017	UF	CT	98.6	1.1	3.83	3.83	859	57.26	3486	25	97	CP152181-CP152182
Ei-110066	2011	UF	DT	98.1	1.0	3.91	3.77	875	56.99	3554	25	96	CP152178-CP152180
Ei-47	2010	DT9	DT	98.4	0.9	3.91	3.77	1292	56.98	3564	25	97	CP152175-CP152177
Ei-39Shipment9	2009	AG1	AG	98.4	1.0	3.80	3.67	849	57.25	3472	25	97	CP152172-CP152174
Ei-40Shipment7	2007	UF	TG	98.4	0.9	3.84	3.67	720	57.26	3510	25	97	CP152168-CP152171
Ei-46	2006	DT8	DT	98.4	0.8	3.78	3.77	934	57.26	3423	25	97	CP152166-CP152167
Ei-45	2006	VL6	VL	98.6	1.0	3.88	3.71	853	57.27	3560	25	97	CP152162-CP152165
Ei-E137	2005	UF	DT	98.6	1.1	3.85	3.71	814	57.26	3529	25	97	CP152158-CP152161
Ei-E126	2004	CT12	CT	98.4	1.8	3.86	3.67	939	57.23	3549	25	97	CP152153-CP152157
Ei-255	2003	VL4	VL	98.4	1.0	3.77	3.73	778	57.45	3472	25	97	CP152148-CP152152
Ei-VN2C02	2002	AG15	AG	98.4	1.0	3.75	3.74	794	57.42	3431	25	97	CP152144-CP152147
Ei-180	2002	AG21	AG	98.6	2.1	3.88	3.74	914	57.31	3556	25	97	CP152140-CP152143
Ei-224	2002	CT24	CT	98.4	1.0	3.80	3.79	859	57.39	3488	25	97	CP152137-CP152139
Ei-31	2001	UF	AG	98.6	1.0	3.85	3.71	966	57.27	3519	25	97	CP152134-CP152136

* UF, unknown farm.

† Provinces include An Giang (AG), Can Tho (CT), Dong Thap (DT), Tien Giang (TG) and Vinh Long (VL).

## Introduction

Vietnam is the leading producer of striped catfish (*Pangasianodon hypophthalmus*), with production volumes totalling 1.5 million tonnes and worth $1.7 billion USD in 2020 [1]. The striped catfish sector is predominantly concentrated within the Mekong Delta region, in An Giang (AG), Can Tho (CT) and Dong Thap (DT) provinces [2]. Catfish farming has been practised in Vietnam since the 1940s, where catfish were traditionally farmed in small, household-owned ponds and were destined for household consumption [3]. The sector underwent a significant transformation in the early 2000s to intensify production and is now dominated by intensive earthen pond culture systems [3]. Intensification of this sector has, however, been accompanied by widespread disease outbreaks from various bacterial pathogens, including *E. ictaluri*, a Gram-negative, rod-shaped bacterium, belonging to the *Hafniaceae* family [4]. The pathogen is the aetiological agent of Bacillary Necrosis of *Pangasius* (BNP) and can cause up to 90% mortality in affected fish populations [5]. In striped catfish, *E. ictaluri* has been described as a facultative intracellular pathogen, surviving and replicating within phagocytes of the fish host [6]. The disease was first reported in farmed striped catfish in Vietnam in 2001 [7,8]. Since then, BNP has persisted as a major challenge for the sector, with recent surveillance suggesting that BNP outbreaks are on the rise [9]. Traditionally, antibiotics, including tetracycline and sulphonamides, have been used for the control of *E. ictaluri* outbreaks in fish farms [10,11]. However, the rapid emergence of antimicrobial resistance (AMR) in *E. ictaluri* populations against several antibiotic classes [8,12,14] now risks treatment failure on the farm, resulting in disease outbreaks that cannot be controlled. This has likely resulted from a long-term overuse and misuse of antibiotics within the Vietnamese aquaculture sector [11,15], driving the acquisition of antimicrobial resistance genes (ARGs) through plasmid-mediated transmission [16], amongst other mobile genetic elements.

The first complete *E. ictaluri* genome was sequenced in 2011, originating from an isolate recovered from channel catfish (*Ictalurus punctatus*) in the USA in 1993 [17]. Since then, only 14 other complete genome assemblies have been deposited in the National Center for Biotechnology Information (NCBI) RefSeq or GenBank databases for comparative analysis (as of 30 June 2024), originating from various hosts in the USA and Southeast Asia (SEA) between 1997 and 2020 (Table S1). Collectively, these published *E. ictaluri* genomes have revealed a genome size of 3.6–4.0 Mb with a G+C of 57.0–57.6% and comprised of a single chromosome with multiple plasmids carrying a diverse set of ARGs and virulence genes [18]. Moreover, the comparative work by others [18], utilizing 11 *E. ictaluri* genomes from various hosts and locations, revealed distinct host-specific genotypes, supporting the findings of earlier studies employing alternative molecular tools [19,21]. Recent comparative genomics research has provided insights into the diversity of *E. ictaluri* circulating within the striped catfish sector in Vietnam, which revealed populations recovered between 2017 and 2021 to be closely related and carry up to three plasmids [22]. Further, the same study also revealed Vietnamese isolates to harbour multiple ARGs against florfenicol, sulphonamides and tetracycline, as well as virulence genes associated with bacterial secretion systems, colicin import and urease activity, amongst others. Whilst this recent study [22] provides valuable data on the genomic diversity in Vietnamese *E. ictaluri*, further genome-wide analysis concerning isolates recovered since BNP was first reported in Vietnam is still needed to explore the genomic variation in isolates causing natural infection over time. We began to explore this in our recent genotyping study utilizing pulsed-field gel electrophoresis, which showed genomic variation in *E. ictaluri* isolates recovered over a 20-year period [23]. Identifying the genomic features behind this variation is now crucial to better understand the drivers of pathogenesis and AMR spread within circulating *E. ictaluri* populations. This will ultimately facilitate the development of improved disease management and control strategies for the Vietnamese striped catfish sector.

In this study, we performed whole-genome sequencing of 31 *E. ictaluri* isolates, recovered from individual disease outbreaks in striped catfish farms across the Mekong Delta region of Vietnam between 2001 and 2021. We then performed comparative genomic analysis to compare the global phylogeny of *E. ictaluri* and explore the population structure and genetic diversity of Vietnamese *E. ictaluri* over time, with a focus on putative plasmids, resistomes and virulomes.

## Methods

### Isolate collection

**Fig. 1.**
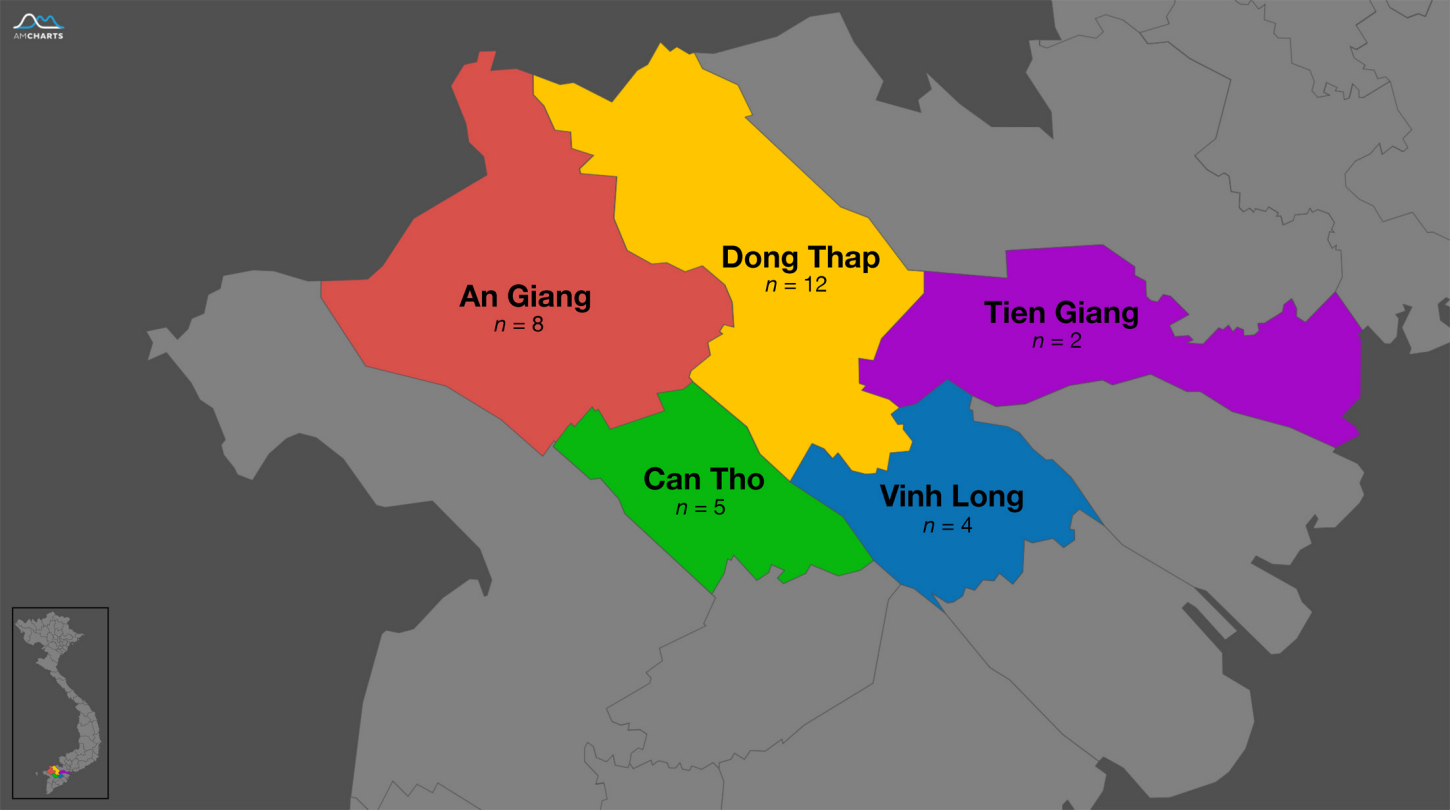
Distribution of *E. ictaluri* isolates recovered from disease outbreaks in *P. hypophthalmus* farms in the Mekong Delta region of Vietnam. Map created using Pixel Map Generator (https://pixelmap.amcharts.com).

The genomes of 31 *E. ictaluri* isolates were sequenced in this study (Table 1). These isolates represented natural disease outbreaks in striped catfish over a 20-year period (2001–2021) and across five provinces within the Mekong Delta region (Fig. 1). All bacteria were isolated from the kidney or liver of fish presenting clinical signs of BNP, including white spots on internal organs, during an active disease outbreak [23]. All *E. ictaluri* isolates were recovered from long-term storage on Protect Beads (SLS, UK) at −80 °C and grown on tryptone soya agar (E and O Laboratories Ltd, UK) at 28 °C for 48 h. In addition to newly sequenced isolates, the representative genome sequences from 15 *E. ictaluri* isolates were retrieved from the NCBI RefSeq database (as of 30 June 2024), where the ‘Complete Genome’ or ‘Chromosome’ level was available (Table S1).

### DNA extraction and purification

All bacteria were grown in 20 ml tryptone soya broth (Oxoid, UK) for 18 h at 28 °C and 140 r.p.m. High molecular weight DNA was extracted using the Wizard® HMW DNA Extraction kit (Promega, UK) following the manufacturer’s instructions, except that 5 µl of RNase A solution was added to the cell lysate and the extracted DNA was eluted in 85 µl of EB buffer (Qiagen, UK). Prior to purification, an additional RNAse treatment was performed with the extracted DNA using RNAse A/T1 (20 µl ml^−1^) (Fisher Scientific, UK), incubated at 37 °C for 30 min. A final purification step was applied using the DNeasy PowerClean Pro kit (Qiagen, UK) following the manufacturer’s instructions with minor modifications, including an additional dry spin at 21 000 ***g*** for 2 min and incubation at 60 °C for 5 min to remove any residual ethanol. Purified DNA was also eluted in 60 µl preheated EB buffer. Purified DNA was quantified using the Qubit dsDNA BR kit (Fisher Scientific, UK) and Qubit machine (Fisher Scientific, UK), before storage at −20 °C, until required.

### Whole-genome sequencing, assembly and annotation

The genomes of isolates were sequenced using the NovaSeq 6000 (Illumina) and MinION Mk1B [Oxford Nanopore Technology (ONT)] platforms at the University of Exeter Sequencing Facility. Long ONT reads were default filtered, and short Illumina reads were adapter- and quality-trimmed using fastp [24]. Hybrid genome assemblies were generated using trimmed ONT and Illumina reads and Unicycler v0.4.8 [25] with default settings on the Bacterial and Viral Bioinformatics Resource Center (BV-BRC) v3.35.5 [26]. Genome quality (e.g. completeness and contamination) was checked by CheckM [27]. Assembled genomes were submitted to GenBank and annotated using the Prokaryotic Genome Annotation Pipeline [28]. The KmerFinder 3.2 database [29,31], hosted on the Center for Genomic Epidemiology (CGE) platform (https://cge.food.dtu.dk/services/KmerFinder/), was employed to confirm the identity of all sequenced isolates. The assembled genomes of sequenced isolates are available at NCBI GenBank via their accession numbers (Table 1).

### Core and pan-genome analysis

The core and pan-genome (core plus accessory genome) of the Vietnamese *E. ictaluri* genomes were determined using the Roary pipeline [32], employing the GFF3 annotation files generated by prokka 1.14.6 [33]. The core genome alignment was used to generate a phylogenetic tree using FastTree v2.1.10 [34], hosted on the Galaxy server (https://usegalaxy.org/). The generated tree and the absence and presence matrix of genes outputted from Roary were used to visualize the pan-genome of *E. ictaluri* using the roary_plots.py script (https://github.com/sanger-pathogens/Roary/blob/master/contrib/roary_plots/roary_plots.py).

### Genome-based phylogeny

The availability of complete sequences of other *E. ictaluri* genomes from various host species and countries allowed for systematic analysis of the evolution of *E. ictaluri* within the global aquaculture sector. First, *in silico* MLST analyses were performed on assembled genomes using PubMLST [35] and the ‘Edwardsiella’ database, which consists of alleles from ten loci: *adk*, *atpD*, *dnaJ*, *gapA*, *glnA*, *hsp60*, *phoR*, *pyrG*, *rpoA* and *tuf*, as described previously [36]. Next, to determine the genetic relatedness between newly sequenced and representative *E. ictaluri* genomes, SNPs were detected using the CSI Phylogeny 1.4 [37] tool available on the CGE platform. The NCBI RefSeq reference genome for *E. ictaluri* (isolate S07-698) was used as the seed genome for the respective SNP calling. An SNP-based maximum-likelihood (ML) phylogenetic tree was built by FastTree 2 [34] and rendered in FigTree v1.4.4 (http://tree.bio.ed.ac.uk/software/figtree/). The population structure of global *E. ictaluri* was defined by SNPs using RheirBAPS, which performs hierarchical clustering of DNA sequence data to reveal nested population structure [38,39]. Three independent iterations were performed with max.depth=2 and upper population sizes of 5, 10 and 15 to obtain optimal clustering of the population. To better understand the evolutionary patterns in point mutations within isolates circulating in Vietnam over the 20-year period, SNP calling was repeated for study isolates using CSI Phylogeny 1.4 and the genome of Ei-31, recovered in 2001, as the reference genome. Finally, average nucleotide identity (ANI) was also calculated for each genome using FastANI [40] and visualized in RStudio 1.4.1717. Here, rows and columns represent each isolate, and the ANI is calculated based on the orthologous mapping of genome sequences.

### Temporal signals and Bayesian phylogenomic analysis

The software SNP-sites [41] was used to generate a suitable alignment of the core genome outputted by Roary using the flag -cb to discard gaps and include monomorphic sites. The final core genome alignment was 2 830 596 bp long. Prior to Bayesian phylogenomic analysis, the temporal signal in the core genome data was determined by TempEst v1.5.3 [42]. Bayesian phylogenetic analysis on the core genome sequence data was performed using BEAST v2.7.7 [43]. The software ModelTest-NG [44,45] was used to determine the most suitable nt substitution model for phylogeny analysis. A generalized time-reversible nt substitution model with a Gamma substitution (four rate categories) and a 0.94 proportion of invariable sites (GTR+Γ4+I) was identified as the best scoring model by the corrected Akaike’s information criterion. The GTR+Γ4+I model was tested in BEAST2 with different clock (strict and optimized relaxed clock) and demographic (constant population size, exponential growth and Bayesian Skyline) parameters to determine the best-fit model. All models were run with a mean clock rate of 1, 50 million Markov chain Monte Carlo (MCMC) states, and with samples taken every 5 000 MCMC states to check for convergence in the sequencing data. Models were compared by Bayes factor of the marginal likelihoods following ‘Nested Sampling’ (NS) estimation with the NS package v1.2.2 [46], applying a particle number of ten (or 50 if results were inconclusive) and a subchain length of 10 000. A final GTR+Γ4+I model with optimized random clock and Bayesian Skyline population dynamics was run independently two times with 100 million MCMC states (sampling every 10 000) and combined using LogCombiner v2.7.7, after having removed a 10% burn-in for each run. Model results were inspected in Tracer to ensure data convergence and optimal modelling, as indicated when all parameters had an effective sample size of >200 (as recommended by BEAST guidelines). A final maximum clade credibility tree to summarize the posterior sample of time-trees was generated using TreeAnnotator v2.7.7 and rendered in FigTree.

### Gene synteny

Gene synteny within chromosome and plasmid sequences was determined by dot plot analysis using MUMmer (v4.0.0rc1-7) [47]. Chromosome or plasmid sequences were first aligned with Nucmer, using the --maxmatch option to include repeat and redundant sequence regions found within both query and reference sequences. The dot plots were then visualized in RStudio with ggplot2 [48], following the script outlined previously [49].

### Accessory genome analysis

Assembled genomes sequenced in this study were inspected for the presence of plasmids by comparing contigs (<1 Mb) against databases comprised of *E. ictaluri* plasmid sequences (*n*=70 sequences, as of 15 April 2024) and the total plasmid sequences available on NCBI (*n*=86 009 sequences, as of 15 April 2024) using blast v2.15.0 [50]. Hits with a similarity threshold >97% and an *E* value of 0 were considered closely related. Further, the assembly graphs generated in BV-BRC were visually inspected with Bandage [51] to determine the structural organization of identified plasmid contigs. Finally, plasmid contigs were screened for plasmid replicon genes using the PlasmidFinder database via ABRicate (https://github.com/tseemann/abricate). A threshold of 60% sequence coverage and 80% sequence identity was set to identify replicon genes [52]. Proksee [53] was used to map features including G+C content and protein CDS of novel plasmids. Analysis of the plasmid pan-genome was conducted using the GView server [54] using default blastn settings. Both chromosome and plasmid sequences were screened for the presence of ARGs by blast analysis against the Comprehensive Antibiotic Resistance Database [55] via ABRicate. A threshold of 60% sequence coverage and 90% sequence identity was set to identify potential ARGs [56]. Homology of amino acid sequences in assembled genomes was searched against the full protein sequence database (setB; *n*=27 982 proteins as of 30 April 2024) of the Virulence Factor Database (VFDB) [57] by blastp using blast v2.15.0. The presence of virulence genes was determined based on a query coverage and sequence identity of 80% [58].

### Statistical analysis

Spearman’s rank correlation coefficient was performed to explore the correlation between two variables. Statistical differences between the means of two variables were determined by a Wilcoxon rank-sum test. A two-tailed Fisher’s exact test was performed to determine the statistical significance of distributions between categorical variables. All statistical analysis was performed on JMP Pro software v17.0.0, with significance determined when *P*<0.05.

## Results

### Genome characteristics

High-quality genome sequences were obtained for all 31 *E. ictaluri* isolates (Table 1). CheckM revealed genomes to have an average 98.51% (98.1–98.6%) completeness and 1.14% (0.8–2.1%) contamination. Genomic features of sequenced isolates were in agreement with the representative *E. ictaluri* genomes available in the NCBI RefSeq database (Table S1). Genome sizes of Vietnamese *E. ictaluri* were on average 3.87 Mb (3.75–4.05 Mb) and comprised of two to five contigs, representing a single chromosome with an average size of 3.76 Mb (3.67–3.92 Mb) and one to four plasmids. Genome coverage ranged from 719× to 1341×, and G+C content ranged from 56.86 to 57.45 mol%. Further, the genomes were comprised on average of 3537 CDS (3423–3739 CDS), 25–28 rRNA genes and 95–98 tRNA genes. All isolates were confirmed as *E. ictaluri* using the KmerFinder database, with a genome query coverage ranging from 89.75 to 98.73%.

### Core and pan-genome

**Fig. 2.**
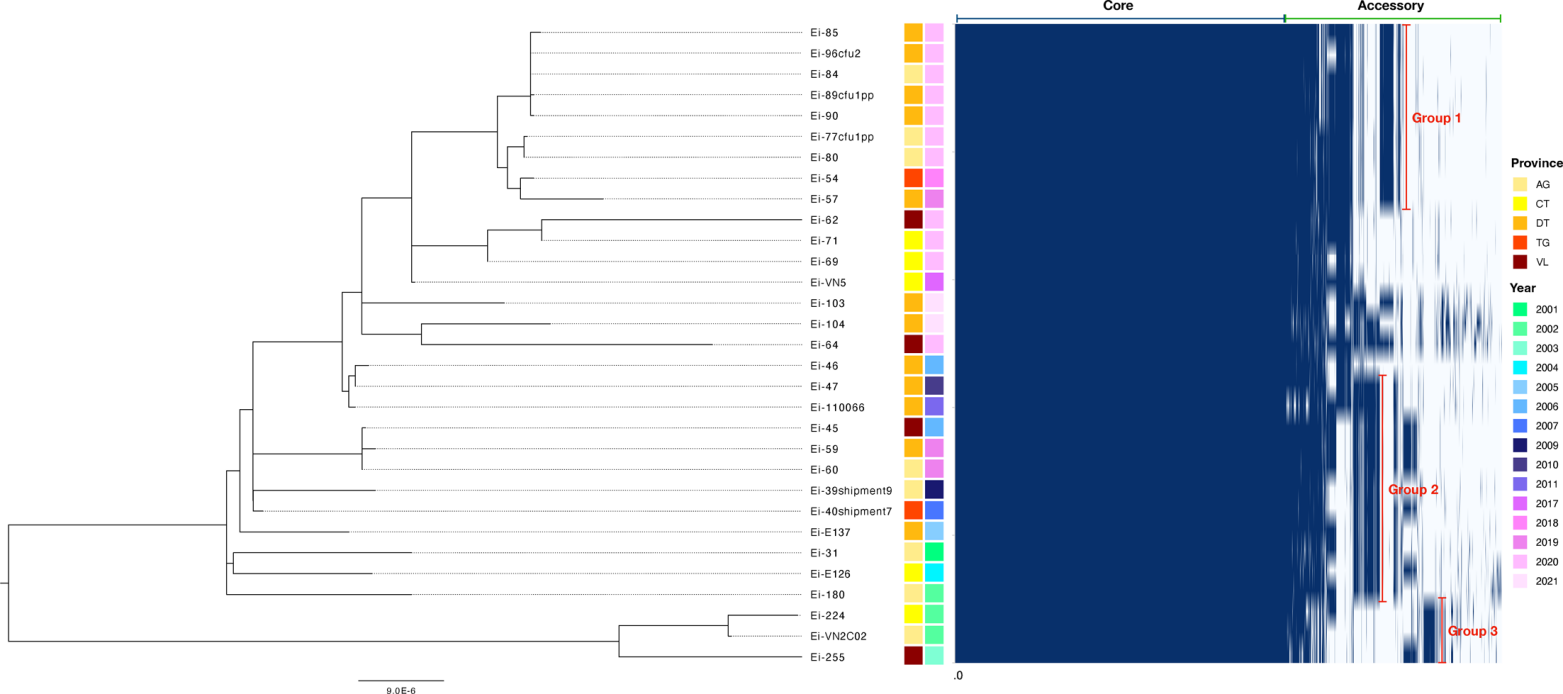
Pan-genome of 31 *E. ictaluri* isolates recovered from *P. hypophthalmus* in Vietnam between 2001 and 2021. The figure shows the gene presence/absence matrix generated from the Roary pan-genome analysis pipeline. Blue and white represent the presence and absence of genes, respectively. Provinces include An Giang (AG), Can Tho (CT), Dong Thap (DT), Tien Giang (TG) and Vinh Long (VL).

A total of 4148 genes were detected in the pan-genome of *E. ictaluri* sequenced in this study (Table S2). The core genome (>96% genomes) accounted for 74% (*n*=3060) of available genes, with a further 98 574 and 416 genes detected in the soft-core (94–95% genomes), shell (13–93% genomes) and cloud (<12% genomes) genomes, respectively. The pan-genome curve did not plateau, indicating an ‘open’ pan-genome (Fig. S1). However, the core genome size stabilized after an initial decrease, where it remained stable at 3060 genes. Isolate Ei-104 contributed the most unique genes (*n*=48) to the pan-genome, followed by Ei-180, Ei-64 and Ei-103, each contributing 30, 29 and 18 unique genes, respectively. Further exploration of the core genome revealed the presence of genes involved in acid resistance (urease; *amtB* and *UreABDFG*), adherence (O-antigen), colicin import (Tol system; *tolABQR*), iron scavenging (*fur*), invasion (invasins and haemolysins), motility (flagellar) and serum resistance (LPS). The dendrogram based on the accessory genome revealed clustering of isolates recovered from different provinces but associated with similar outbreak years (Fig. 2). Three distinct groupings were identified according to the accessory genome, where isolates harboured a unique collection of genes often associated with either AMR, bacterial toxins or virulence mechanisms. Isolates in group 1, recovered between 2018 and 2020, were found to carry genes encoding for beta-lactam resistance (*blaTEM*) and a flagellar biosynthetic protein (*flhB*). Isolates in group 2 were predominantly recovered between 2001 and 2011 and carried resistance genes against aminoglycoside (*neo*) and arsenic (*ars*-family) compounds. Finally, isolates within group 3, recovered between 2002 and 2003, carried unique genes encoding for cellular contact-dependent growth inhibition (*cdiA_1*) as well as those involved in a *Salmonella* pathogenicity island (*dnaB-PI*) and type IV secretion system (T4SS).

### Genome-based phylogeny

*In silico* MLST profiling revealed sequence type (ST) 26 to be the predominant ST circulating the Vietnamese striped catfish sector since 2001, where this ST accounted for 94% (*n*=29) of genomes sequenced in this study (Table S3). Further, ST 26 was the most prevalent ST assigned to *E. ictaluri* isolates from Asia, accounting for 92% (*n*=35) of genomes recovered from various fish species in China, Japan, Thailand and Vietnam. In contrast, the *E. ictaluri* genomes from isolates recovered from *Ictalurus* catfish species and zebrafish (*Danio rerio*) in the USA were distinct from those from Asia, being assigned to either ST 19, 20 or 23. Both genomes associated with Nile tilapia (*Oreochromis niloticus*) were assigned to ST 24, irrespective of country. The allelic profiles for isolates Ei-103 and Ei-180 sequenced in this study were unique to the PubMLST database and therefore classified as novel STs. On closer inspection, these isolates shared similar profiles to ST 26, except that they differed in alleles for *rpoA* and *adk*, respectively.

**Fig. 3.**
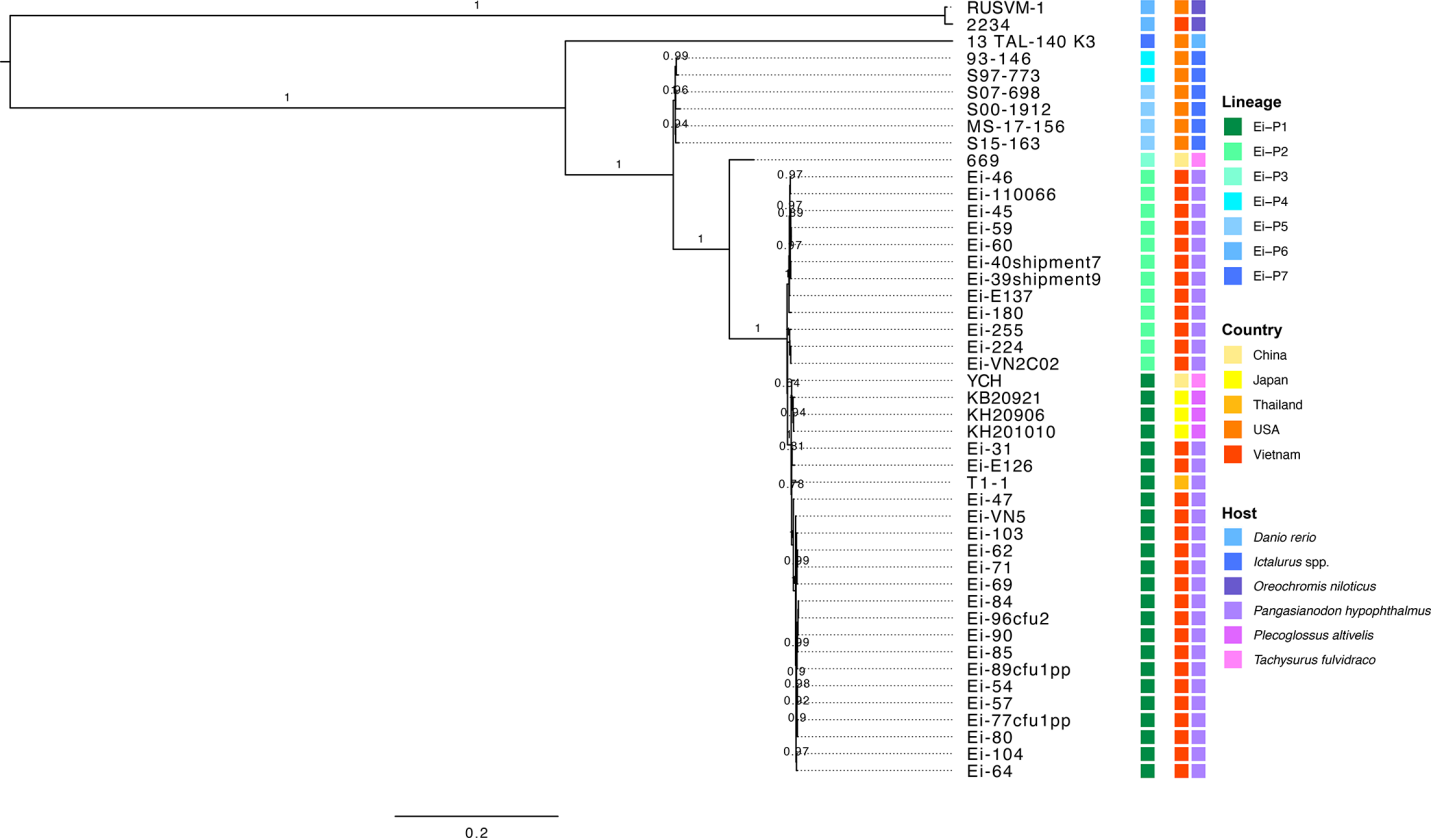
Population structure of *E. ictaluri* isolates from various aquatic sources. ML phylogenetic tree of 46 *E. ictaluri* genomes based on 8 175 high-quality SNP positions. The genome of *E. ictaluri* S07-698 was used as a reference sequence for SNP detection. Bootstrap values >0.6 are highlighted on selected branches. Lineage, country and host origin for each isolate are provided at the same horizontal position. Host species designated as *Ictalurus* spp. include *Ictalurus furcatus*, *I. punctatus* and hybrid catfish (*I. furcatus* x *I. punctatus*).

A total of 8 175 high-quality SNP loci were detected across the global *E. ictaluri* genome collection. Based on phylogenetic analysis, all *E. ictaluri* were clustered into seven lineages, designated Ei-P1 to Ei-P7 (Fig. 3). Fifty-two percent (*n*=24) of isolates were assigned to Ei-P1. Exploration of *E. ictaluri* lineages with country and host-level data revealed all isolates from Ei-P1 to be predominantly represented by isolates recovered from striped catfish in Vietnam, although isolate T1-1 (striped catfish; Thailand) and isolates from ayu (*Plecoglossus altivelis*) and yellowhead catfish (*Tachysurus fulvidraco*) in Japan and China, respectively, also belonged to this lineage. The other lineages were found to be country- or host-specific. Both Ei-P1 and Ei-P2 were found to be widely dispersed within Vietnam during the past 20 years. The lineage Ei-P1 has been circulating since 2001, whilst Ei-P2 was first detected in 2002. Temporal variation was also noted in the prevalence of these two lineages in Vietnam between 2001 and 2021. Whilst the lineage Ei-P2 was more dominant between 2002 and 2009, Ei-P1 became frequently detected by 2017 and was the dominant lineage circulating in Vietnam by 2020. The ML tree constructed from all SNP loci revealed isolates sequenced in this study to be closely related, where they differed by less than 185 SNP positions (mean: 53). Further, several isolates from different provinces even showed high genome similarity (zero SNP differences). These isolates were recovered from AG and DT in 2019 (Ei-60 and Ei-59), as well as several provinces in 2020, including CT and VL (Ei-71 and Ei-62) and, lastly, AG and DT (Ei-84 and Ei-96cfu2). When evolutionary patterns in point mutations were explored for sequenced Vietnamese isolates, 48 SNPs were found to be associated with specific outbreak periods (Table 2). The majority of SNPs (75%; *n*=36) were detected in isolates recovered between 2002 and 2003. On further exploration, SNPs were detected in genes involved in antibiotic resistance or virulence pathways, including *ampE* and *fliQ*, respectively. Further point mutation events in the *E. ictaluri* genome were also detected in 2005, 2006 and 2017, where the SNPs persisted in >89% of isolate genomes recovered up to 2021. Amongst these mutation events, SNPs in the virulence gene *flgK* and the quinolone target *parC* were detected within the genome of all isolates recovered after 2005 and 2006, respectively.

**Table 2.** Unique SNP detected in the genomes of *E. ictaluri* recovered from *P. hypophthalmus* in Vietnam between 2002 and 2021. The SNPs were called against the genome of Ei-31, recovered from AG province in 2001

Gene	Annotation	SNP	Outbreak years	No. of isolates (%)
AAGU61_00085	Cytochrome o ubiquinol oxidase subunit III	T>C	2002–2003	3 (75)
AAGU61_02185	*UbiX* family flavin prenyltransferase	T>C	2002–2003	3 (75)
AAGU61_07575	Acyltransferase	A>G	2002–2003	3 (75)
AAGU61_08365	Class I adenylate cyclase	C>T	2002–2003	3 (75)
AAGU61_09015	*AraC* family transcriptional regulator	G>A	2002–2003	3 (75)
AAGU61_09310	*YfdX* family protein	T>C	2002–2003	3 (75)
AAGU61_09315	Transcriptional regulator	T>C	2002–2003	3 (75)
AAGU61_10185	*NupC/NupG* family nucleoside CNT transporter	G>A	2002–2003	3 (75)
AAGU61_10230	Cytochrome b/b6 domain-containing protein	G>A	2002–2003	3 (75)
AAGU61_10970	Peptidoglycan glycosyltransferase f*tsI*	A>G	2002–2003	3 (75)
AAGU61_11180	PTS sugar transporter subunit IIA	T>C	2002–2003	3 (75)
AAGU61_12255	ATP-binding cassette domain-containing protein	A>T	2002–2003	3 (75)
AAGU61_12275	Sodium:solute symporter	A>C	2002–2003	3 (75)
AAGU61_13935	Transketolase	G>A	2002–2003	3 (75)
AAGU61_14395	M20 family metallo-hydrolase	C>A	2002–2003	3 (75)
AAGU61_15325	HI1450 family dsDNA-mimic protein	C>T	2002–2003	3 (75)
AAGU61_15990	Mechanosensitive ion channel domain-containing protein	C>T	2002–2003	3 (75)
AAGU61_16040	Hypothetical protein	A>T	2002–2003	3 (75)
*ampE*	Beta-lactamase regulator *ampE*	A>G	2002–2003	3 (75)
*bcp*	Thioredoxin-dependent thiol peroxidase	A>G	2002–2003	3 (75)
*dtpA*	Dipeptide/tripeptide permease d*tpA*	G>T	2002–2003	3 (75)
*envZ*	Two-component system sensor histidine kinase e*nvZ*	A>G	2002–2003	3 (75)
*fliQ*	Flagellar biosynthesis protein f*liQ*	C>T	2002–2003	3 (75)
*hyaA*	Hydrogenase 1 small subunit	A>C	2002–2003	3 (75)
*ilvD*	Dihydroxy-acid dehydratase	G>C	2002–2003	3 (75)
*kduI*	5-Dehydro-4-deoxy-d-glucuronate isomerase	T>C	2002–2003	3 (75)
*mutL*	DNA mismatch repair endonuclease m*utL*	C>T	2002–2003	3 (75)
*nfsA*	Oxygen-insensitive NADPH nitroreductase	G>T	2002–2003	3 (75)
*nrfB*	Cytochrome c nitrite reductase pentaheme subunit	C>T	2002–2003	3 (75)
*pgsA*	CDP-diacylglycerol-glycerol-3-phosphate 3-phosphatidyltransferase	G>A	2002–2003	3 (75)
*phsA*	Thiosulphate reductase *phsA*	T>A	2002–2003	3 (75)
*rpoC*	DNA-directed RNA polymerase subunit beta	T>C	2002–2003	3 (75)
*rpoD*	RNA polymerase sigma factor *rpoD*	C>T	2002–2003	3 (75)
*rpoH*	RNA polymerase sigma factor *rpoH*	A>G	2002–2003	3 (75)
*sapD*	Putrescine export ABC transporter ATP-binding protein s*apD*	C>T	2002–2003	3 (75)
*tilS*	tRNA lysidine [34] synthetase t*ilS*	C>A	2002–2003	3 (75)
AAGU61_05240	Sulphatase-like hydrolase/transferase	G>A	2005–2021	25 (100)
*flgK*	Flagellar hook-associated protein *flgK*	G>A	2005–2021	25 (100)
AAGU61_03580	Invasin domain 3-containing protein	C>T	2006–2021	25 (100)
*dcuC*	C4-dicarboxylate transporter *dcuC*	G>A	2006–2021	25 (100)
*parC*	DNA topoisomerase IV subunit A	G>T	2006–2021	25 (100)
*sapF*	Putrescine export ABC transporter ATP-binding protein s*apF*	C>A	2006–2021	25 (100)
*aaeB*	p-Hydroxybenzoic acid efflux pump subunit *AaeB*	G>T	2017–2021	16 (89)
AAGU61_03885	*Ail/Lom* family outer membrane beta-barrel protein	C>A	2017–2021	16 (89)
AAGU61_16180	Oligopeptidase B	G>A	2017–2021	16 (89)
*glgC*	Glucose-1-phosphate adenylyltransferase	C>T	2017–2021	16 (89)
*ptsH*	Phosphocarrier protein *Hpr*	G>A	2017–2021	16 (89)
*purE*	5-(Carboxyamino)imidazole ribonucleotide mutase	C>T	2017–2021	16 (89)

**Fig. 4.**
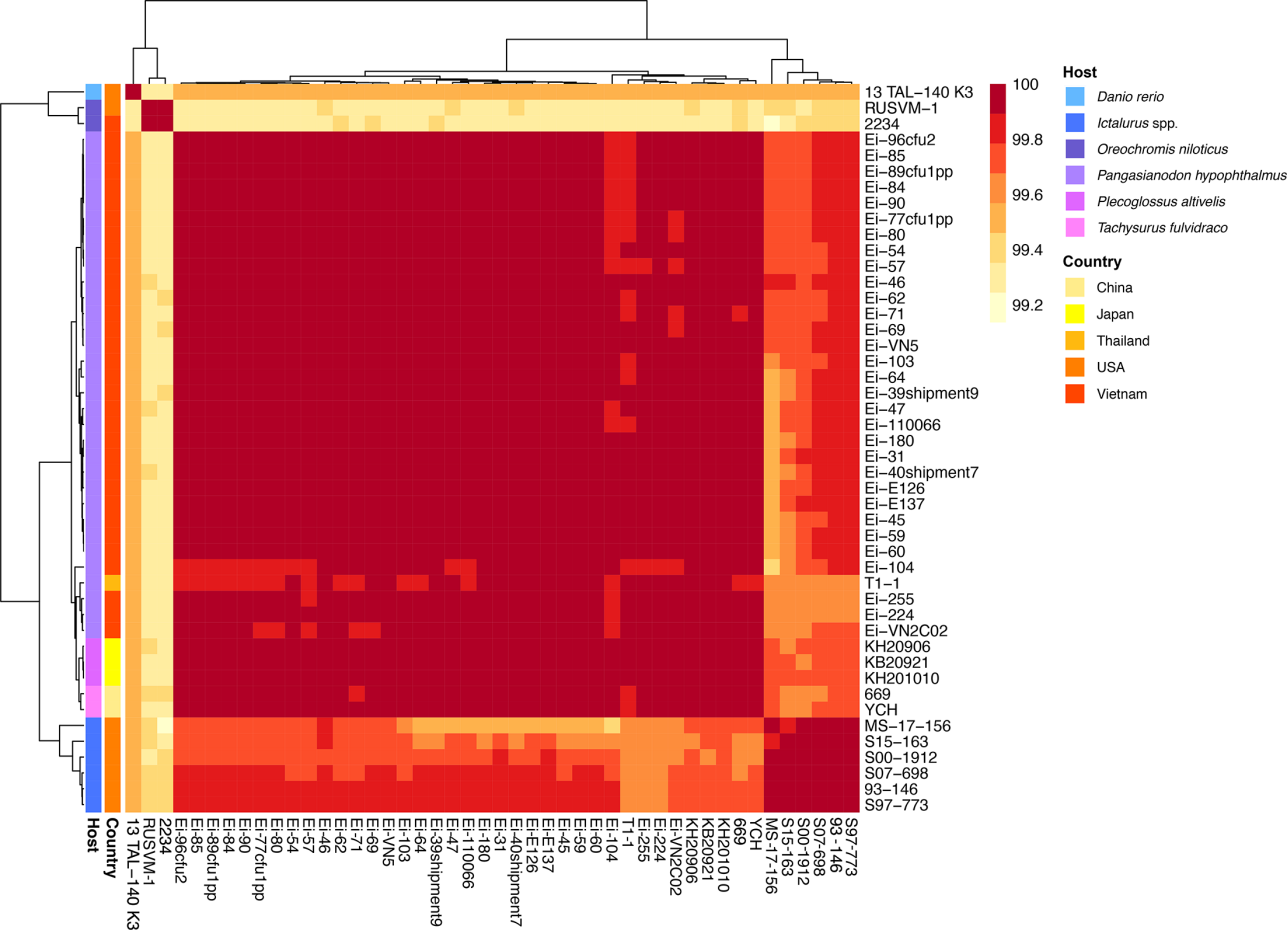
Heatmap of the ANI of 46 *E. ictaluri* isolates from various aquatic sources. The pairwise ANI values are provided in Table S4. The metadata (country and host origin) for each isolate is provided at the same horizontal position. Host species designated as *Ictalurus* spp. include *I. furcatus*, *I. punctatus* and hybrid catfish (*I. furcatus* x *I. punctatus*).

ANI of Vietnamese isolates sequenced in this study revealed genomes to share >99.86% sequence similarity (Fig. 4). Further, the global *E. ictaluri* genome collection shared >99.14% similarity based on ANI. Analysis of the ANI-based phylogeny revealed distinct host-associated genotypes for isolates recovered from *Ictalurus* catfish species, Nile tilapia and zebrafish. However, isolates recovered from ayu, striped catfish and yellowhead catfish across Asia were found to form a single clade, where isolates shared >99.86% genome similarity.

### Temporal signals and Bayesian phylogenomic analysis

**Fig. 5.**
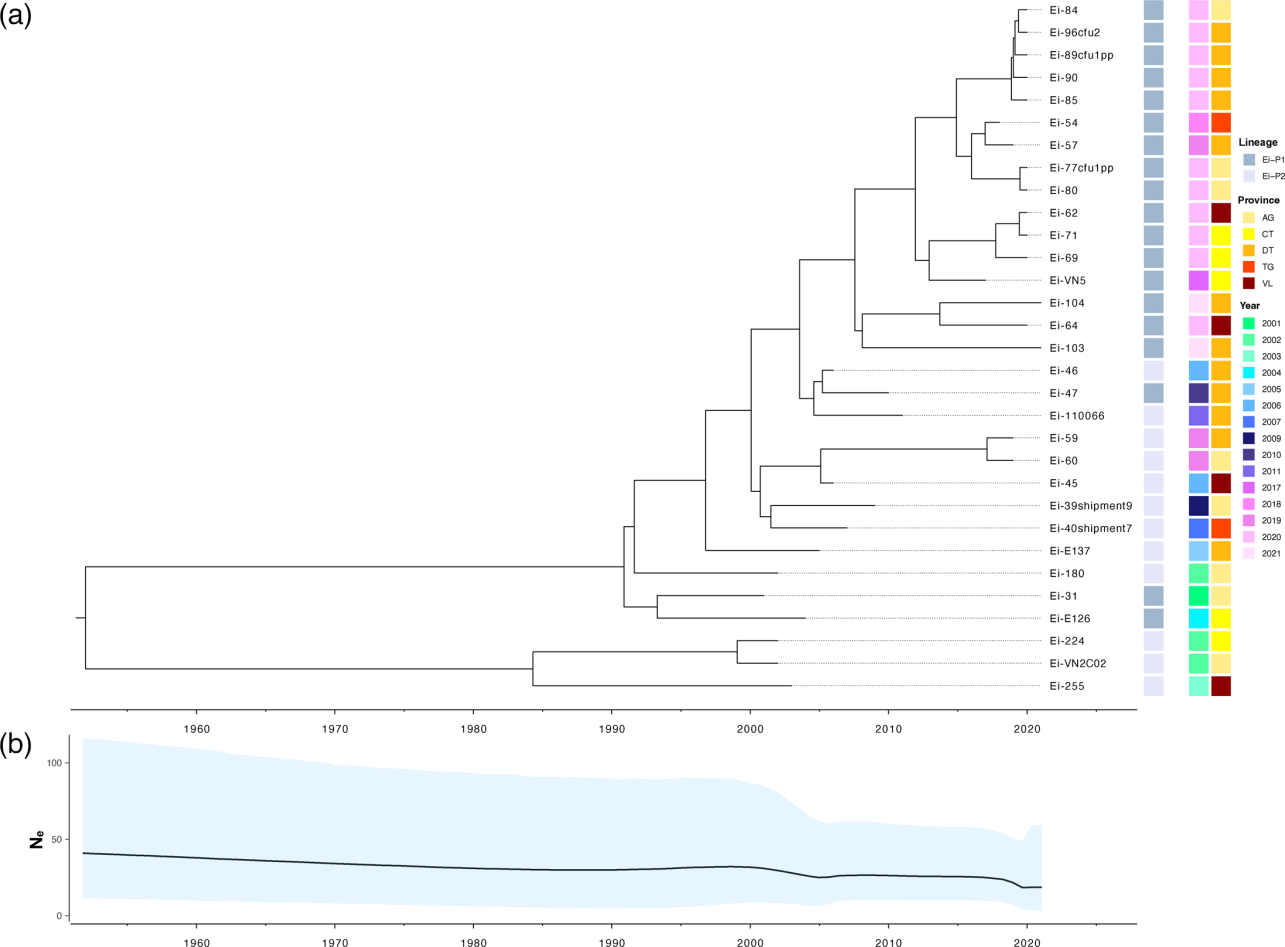
Bayesian analysis of *E. ictaluri* lineages circulating in Vietnam between 2001 and 2021. An MCMC tree showing the time-scaled phylogeny of *E. ictaluri* obtained using the BEAST tool. Time-scaled phylogeny was performed on a core genome alignment of 2 830 596 sites, using a Bayesian Skyline population demographic model and optimized relaxed clock (**a**). Provinces include An Giang (AG), Can Tho (CT), Dong Thap (DT), Tien Giang (TG) and Vinh Long (VL). A Bayesian Skyline plot showing demographic changes measured as effective population size (*N*_*e*_) per generation (**b**). Upper and lower *N*_*e*_ values, as estimated by BEAST, are illustrated in blue.

The core genome data were found to have sufficient temporal signal for time-scaled phylogeny, with a correlation coefficient and *R*^2^ of 0.85 and 0.72, respectively, for the dated tips. Bayesian analysis of the core genome revealed that the most recent common ancestor (MRCA) for *E. ictaluri* circulating in Vietnam was around 1951 [95% highest probability density (HPD): 1886–1996] (Fig. 5a). Moreover, populations rapidly diverged into two main lineages, which were dominated by an ancestral lineage of Ei-P2 until the 1990s. By 1991–1992, ancestral lineages of Ei-P1 and Ei-P2 were both circulating in the striped catfish sector in Vietnam. However, the MRCA for most Ei-P1 isolates did not emerge until around 2003. The populations circulating the sector in 2021 diverged from their MRCA around 2007. The evolutionary rate for Vietnamese *E. ictaluri* was estimated at 1.468×10^−6^ substitutions per site per year (95% HPD: 2.5136×10^−6^–6.2175×10^−7^), which corresponded to a mutation rate of 4.2 sites per genome per year. The effective population size of *E. ictaluri* in Vietnam showed a decline between 1951 (mean *N*_*e*_=40.95) and 2021 (mean *N*_*e*_=18.60). Whilst there was a steady decline for the first five decades (*N*_*e*_ difference=10.62), a much more rapid decline was inferred by BEAST between 2001 (mean *N*_*e*_=30.33) and 2005 (mean *N*_*e*_=25.25) and again between 2017 (mean *N*_*e*_=24.47) and 2021 (Fig. 5b).

### Chromosome sequence analysis

**Fig. 6.**
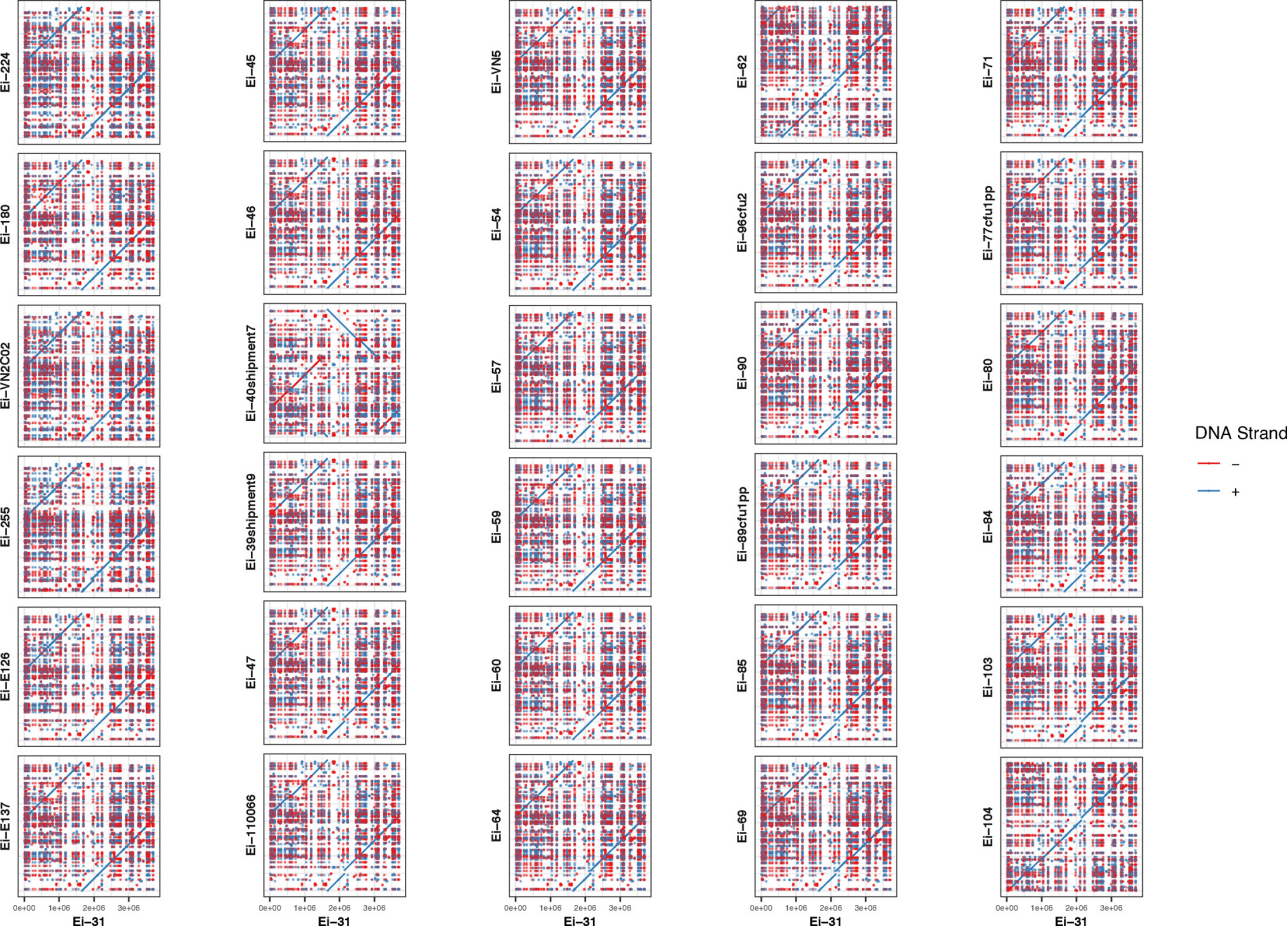
Pairwise genome alignment of *E. ictaluri* isolates recovered from *P. hypophthalmus* in Vietnam between 2001 and 2021. Dot plots of isolates are ordered by year from top left (2002) to bottom right (2021). The genome of Ei-31, recovered in 2001, was used as the reference genome and aligned with query genomes using MUMmer. Dot plots were generated in RStudio using ggplot2 (see methods).

Synteny analysis of chromosome sequences revealed that whilst there was a high degree of gene order conservation amongst genomes of *E. ictaluri* circulating in Vietnam between 2001 and 2021, large-scale genomic rearrangements were evident in isolates recovered after 2002 (Fig. 6). These included duplications and relocations in 97% (*n*=29) of the genomes sequenced. The chromosome of Ei-40shipment7 was also characterized by a large region of inversion in reference to Ei-31 and not evident in other genomes. The dot plot of the Ei-104 chromosome revealed a high degree of gene synteny with Ei-31.

### Plasmid sequence analysis

**Fig. 7.**
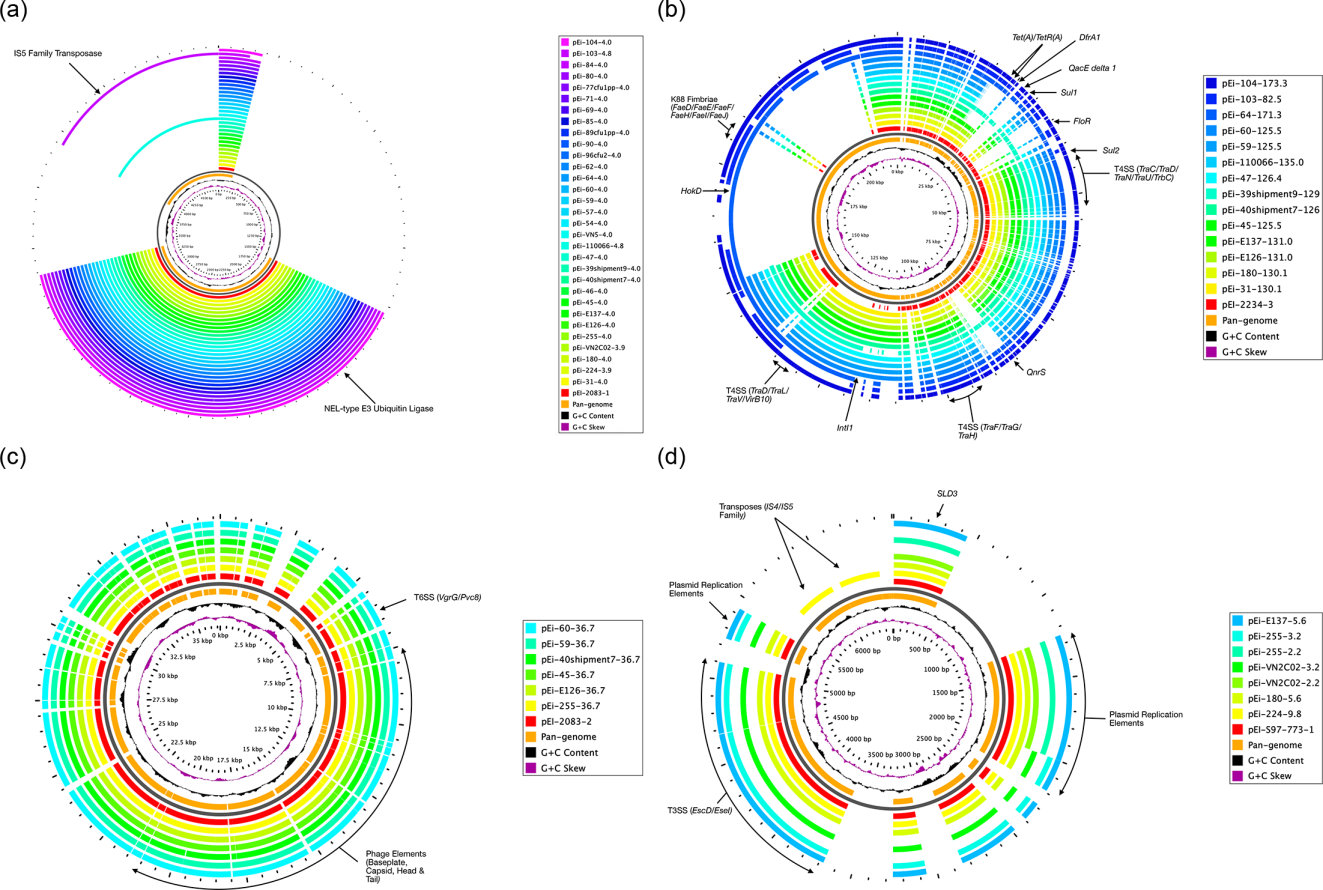
Pan-genome analysis of *E. ictaluri* plasmids detected by whole genome sequencing in this study. Pan-genome analysis was performed for the 4.0 (**a**), 82.5–173.3 (**b**), 36.7 (**c**) and 2.2–9.8 kb (**d**) plasmids. The innermost circle represents the pan-genome (orange), and the outer circles indicate the plasmids detected in this study along with the reference plasmid (red).

Isolates harboured one to four putative plasmids, ranging in size from 2.2 to 173.3 kb (Table S5). A single conserved plasmid (4.0–4.8 kb) was detected in all isolates sequenced. Dot plot analysis revealed a high degree of gene synteny for this plasmid across the genome collection, although there was evidence of gene duplication in some genomes (Fig. S2). Further, this plasmid was identical to pEI-2083–1 (4.0 Kb), previously detected in *E. ictaluri* isolate T1-1 and recovered from striped catfish (100% coverage and 100% identity). This was supported by results from blast analysis of the plasmid pan-genome, which also highlighted the presence of a conserved ubiquitin ligase gene (Fig. 7a). A large (82.5–173.3 kb) IncA/C2_1-type plasmid was also frequently detected in *E. ictaluri* circulating in Vietnam since 2001 (*n*=14; 45%). However, whilst there was a high degree of gene synteny in the IncA/C2_1-type plasmid in *E. ictaluri* between 2001 and 2005, extensive plasmid rearrangement was observed in the plasmid of isolates recovered after 2006, including deletions, duplications and inversions (Fig. S3). Analysis of the plasmid pan-genome showed a similar backbone (46–86% coverage and >97% identity) to plasmid pEI-2234–3 (109.5 kb) found in *E. ictaluri* isolate 2234 and recovered from Nile tilapia (Fig. 7b). A 36.7 kb plasmid, closely related to *E. ictaluri* T1-1 plasmid pEI-2083–2 (36.7 kb; 100% coverage and >99% identity), was also identified in a small group of isolates (*n*=6; 19%), which first emerged in 2003. Both dot plot and blast analysis demonstrated high genomic relatedness between the plasmids, although minor gene rearrangement (insertions) was noted between the respective genomes (Figs 7c and S4). Five (16%) isolates, recovered between 2002 and 2005, were found to harbour a small (2.2–9.8 kb) plasmid. Both dot plot and blast analysis revealed low gene synteny and relatedness between the plasmids, although all plasmids shared >91% coverage and >99% identity with pEI-S97-773-1, which was recovered from a channel catfish-associated *E. ictaluri* isolate in USA (Figs 7 and S5). Moreover, both pEi-180–5.6 and pEi-E137-5.6 had identical backbones to pEI-S97-773-1.

**Fig. 8.**
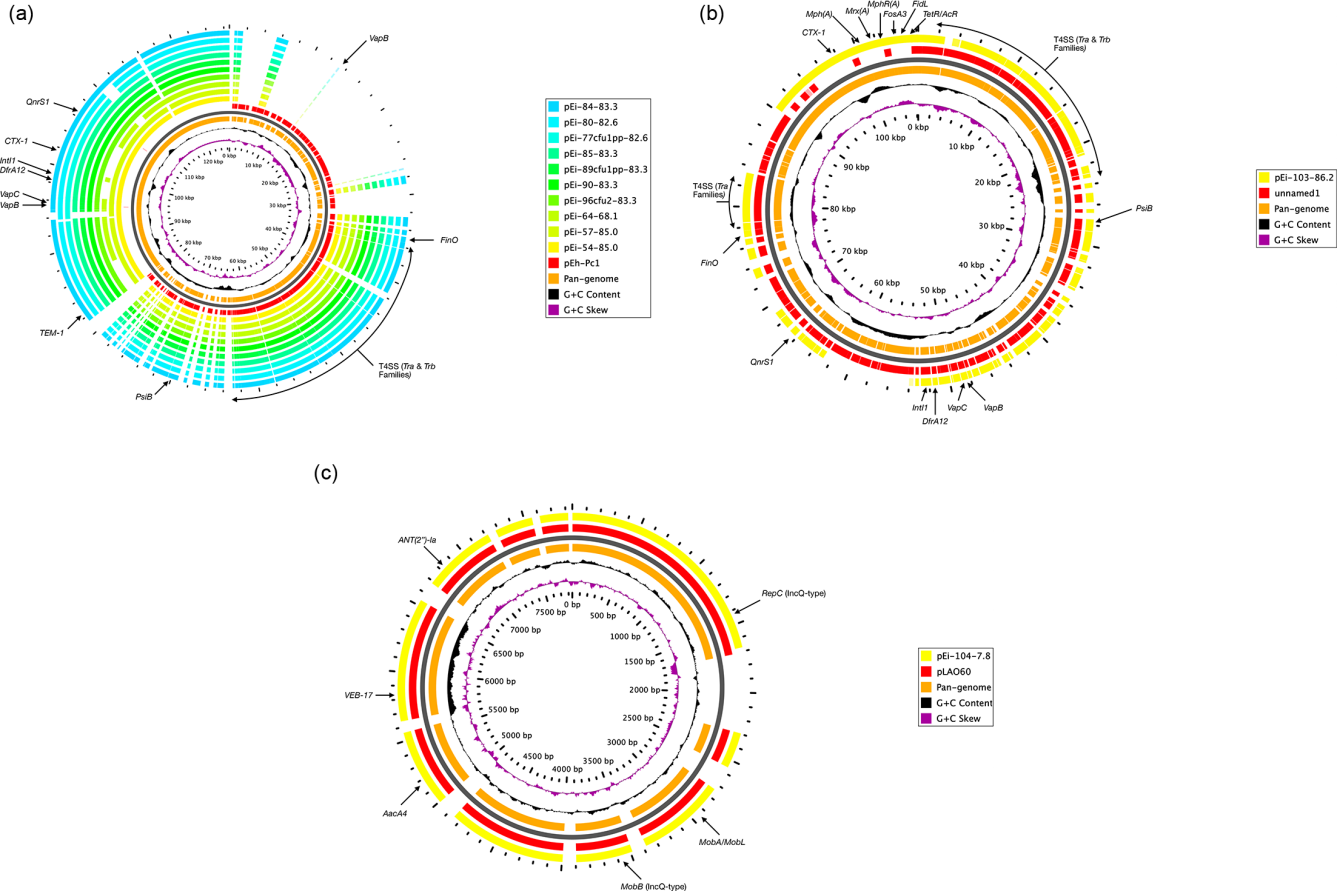
Pan-genome analysis of *E. ictaluri* plasmids detected by whole-genome sequencing in this study. Pan-genome analysis was performed for the 68.1–85.0 kb (**a**), pEi-103-86.2 (**b**) and pEi-104–7.8 (**c**) plasmids. The innermost circle represents the pan-genome (orange), and the outer circles indicate the plasmids detected in this study along with the reference plasmid (red).

**Fig. 9.**
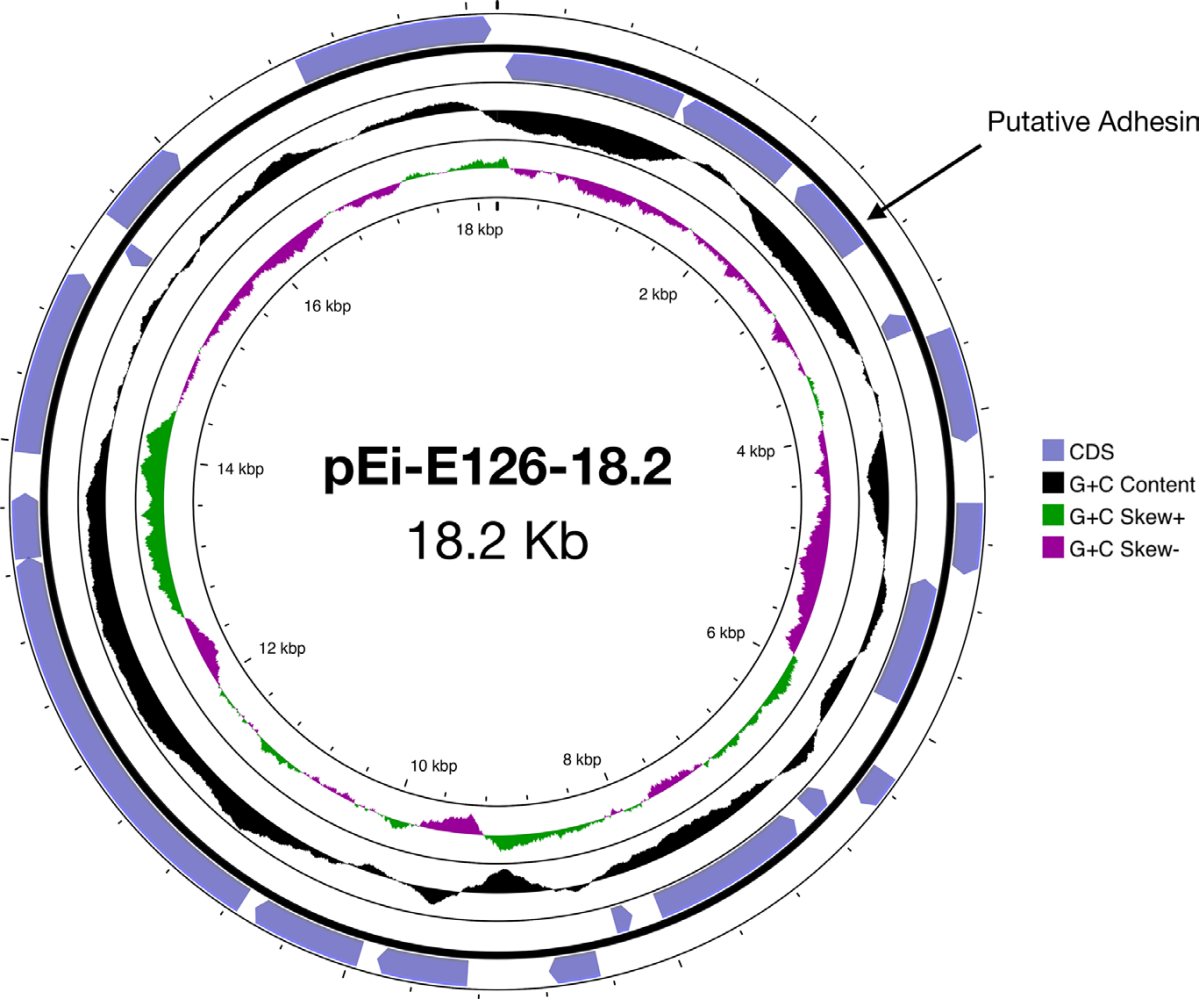
Map of novel 18.2 kb plasmid detected in *E. ictaluri* isolate Ei-E126. Protein CDS regions, G+C content and skew visualized using Proksee.

Plasmid reconstructions also detected the presence of a large (68.1–86.2 kb) plasmid in 66% (*n*=10) isolates, recovered between 2018 and 2020. These plasmids were shown to have high gene synteny, although plasmid rearrangement was observed after 2019 with inversions frequently noted in the genomes of isolates recovered in 2020 (Fig. S6). Moreover, the plasmids were closely related to pEh-Pc1 from *Edwardsiella tarda* (55–68% coverage and 97% identity; Table S5), with conserved genes encoding for type II toxin-antitoxin system, virulence determinants and elements of F-type plasmids (e.g. *FinO* and *PsiB*) (Fig. 8a). Two unique plasmids were identified in isolates Ei-103 and Ei-104, with sizes of 86.2 and 7.8 kb, respectively. Plasmid pEi-103–86.2 was closely related to a 90.2 kb plasmid in *Escherichia coli* (79% coverage and 98% identity), with conserved AMR, virulence and F-type plasmid genes (Fig. 8b). Moreover, plasmid pEi-104–7.8 was confirmed as an IncQ-type plasmid, which had an identical backbone (100% coverage and 99% identity) to pLAO60 in *E. coli* (Fig. 8c). Isolate Ei-E126 was found to carry a novel 18.2 kb plasmid with a G+C content of 50.73 mol%. Plasmid pEi-E126-182 comprised of 21 CDS primarily encoding hypothetical proteins and insertion sequences; however, a putative adhesin was detected that was identical to adhesins previously detected in *E. ictaluri* (data not shown) (Fig. 9).

### Resistome

**Fig. 10.**
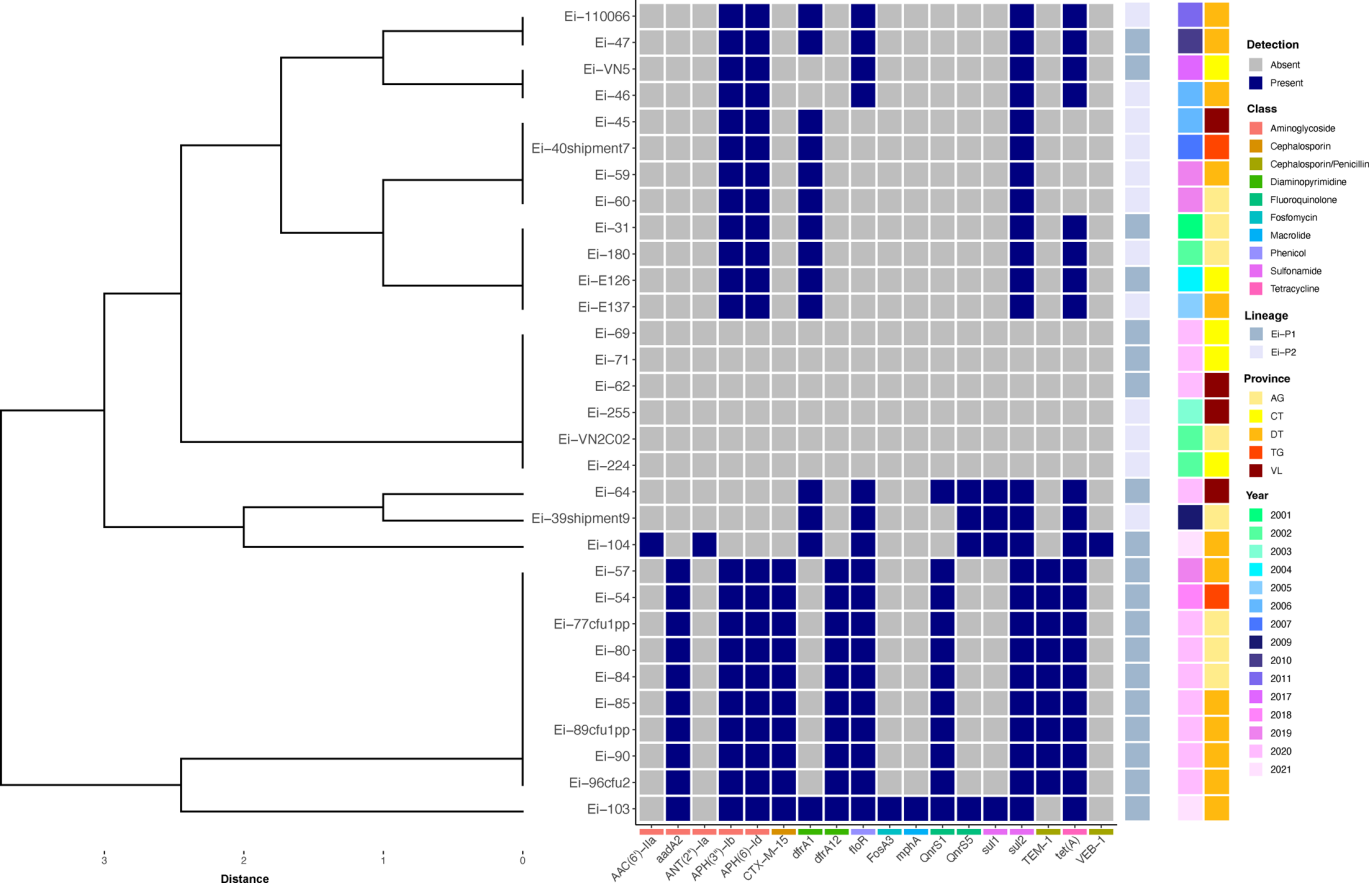
Heatmap of antimicrobial resistance genes detected in 31 *E. ictaluri* genomes recovered from *P. hypophthalmus* in Vietnam between 2001 and 2021. Dendrograms were generated using Euclidean distance and unweighted pair group method with arithmetic mean. Provinces include An Giang (AG), Can Tho (CT), Dong Thap (DT), Tien Giang (TG) and Vinh Long (VL).

A total of 18 ARGs were detected across 81% (*n*=25) genomes sequenced in this study (Fig. 10). Further, these ARGs were categorized into either antibiotic efflux (*n*=2; 11%), antibiotic inactivation (*n*=10; 56%), antibiotic target protection (*n*=2; 11%) and antibiotic target replacement (*n*=4; 22%) and encoded resistance to ten antibiotic classes including aminoglycosides, cephalosporins, diaminopyrimidines, fluoroquinolones, fosfomycins, macrolides, penicillins, phenicols, sulphonamides and tetracyclines. Further exploration of annotated genomes revealed six ARGs [*APH(3'')-Ib*, *APH (6)-Id*, *dfrA1*, *floR*, *sul2* and *tet(A*)] to be distributed across chromosome or plasmid sequences, whilst the remaining 12 ARGs were exclusive to plasmids only. The *sul2* gene, encoding resistance to sulphonamides, was the most prevalent ARG in *E. ictaluri* genomes (*n*=25; 81%). Moreover, in 56% (*n*=14) of *sul2*-carrying isolates, the ARG was found to be conserved on the pEI-2234–3-like plasmid (Fig. 7b). Aminoglycoside and tetracycline ARGs were also highly prevalent with 22 (71%), 22 and 21 (68%) genomes found to harbour *APH(3’)-lb*, *APH (6)-ld* and *tet(A*) genes, respectively. Additionally, these ARGs were also located on 71% (*n*=10), 71% and 64% (*n*=9) of the pEI-2234–3-like plasmids carried by *E. ictaluri* in this study, respectively (Fig. 7b). Fosfomycin and macrolide resistance genes were less frequently found. Resistance mechanisms against aminoglycosides were the most diverse with five ARGs detected across the genome collection, although all genes fell into the antibiotic inactivation category. Lineage Ei-P1 harboured significantly more ARGs than Ei-P2 (Wilcoxon, *P*<0.001); up to 14 (average 7±1 genes) ARGs were detected in isolates from Ei-P1, whereas up to six (average 4±1 genes) ARGs were detected in isolates from Ei-P2. Moreover, the presence of the ARGs *aadA2*, *CTX-M-15*, *dfrA12*, *floR*, *QnrS1*, *tet(A*) and *TEM-1* was found to be significantly associated with lineage Ei-P1 (two-tailed Fisher’s exact test, *P*<0.05).

**Fig. 11.**
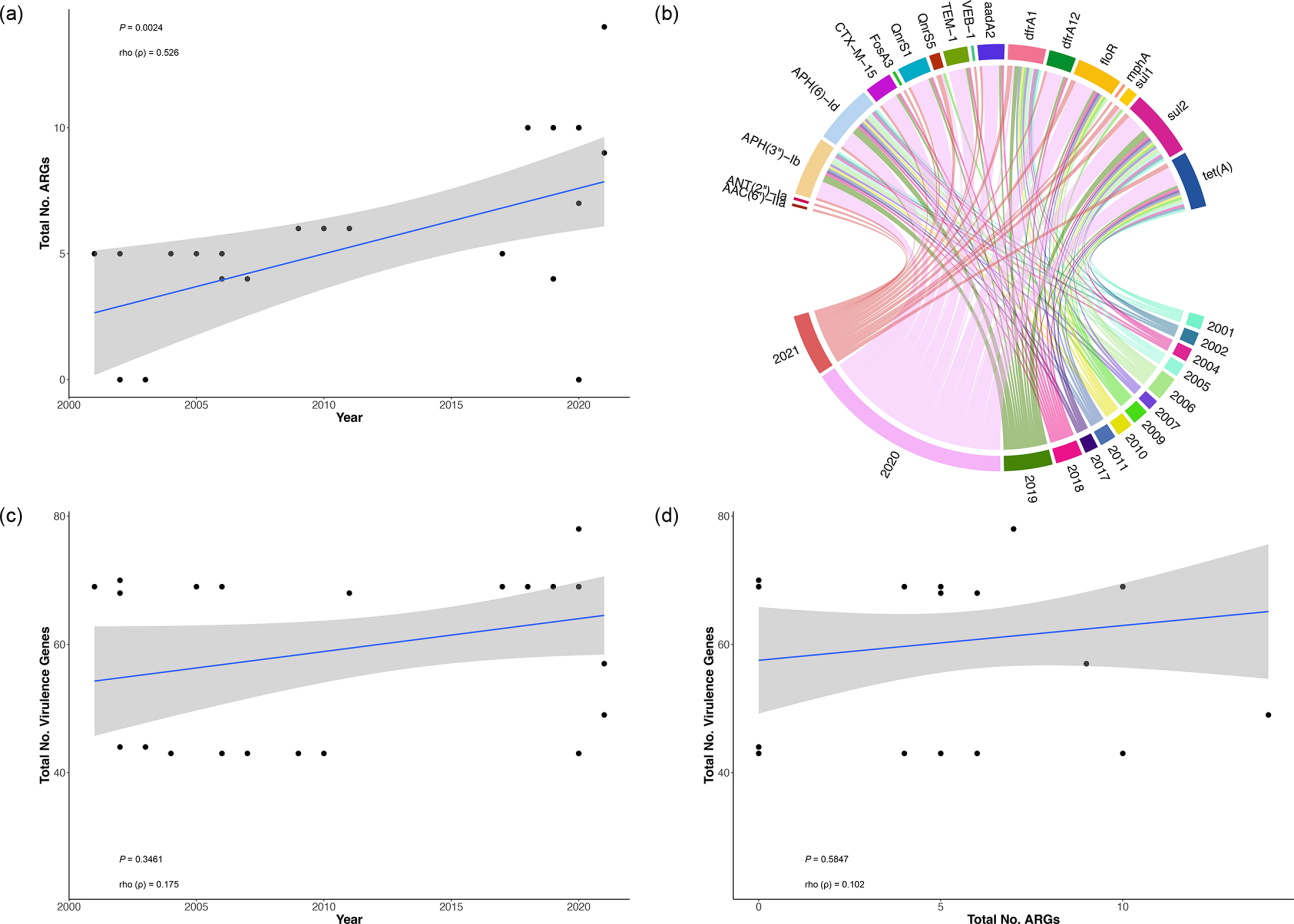
Changes in antimicrobial resistance gene (ARG) and virulence genes in Vietnamese *E. ictaluri* over time. The correlation of ARG number with isolate recovery times (a). Chord diagram showing the various ARGs detected in genomes by outbreak year (b). The correlation of virulence gene number with isolate recovery times (c). The correlation between ARG and virulence gene in different isolates (d).

Results from correlation analysis showed that the number of ARGs detected in *E. ictaluri* genomes from Vietnam increased significantly in association with the isolate’s recovery time (Spearman’s ρ, *P*<0.01) (Fig. 11a). The number of ARGs detected in *E. ictaluri* genomes increased from five in 2001 (Ei-31) to 14 in 2021 (Ei-103). Several ARGs were found to have persisted within *E. ictaluri* populations over the 20-year time span, including *APH(3’)-lb*, *APH (6)-ld*, *dfrA1*, *sul2* and *tet(A*) (Fig. 11b), and are likely associated with the pEI-2234–3-like plasmids also circulating in Vietnam in the same period (Fig. 7b). However, resistance to certain antibiotic classes in *E. ictaluri* was found to be acquired over time. This included resistance to fluoroquinolones, encoded by *QnrS1*, which was absent in the genomes of isolates recovered before 2009 but became prevalent by 2018. Moreover, this trend was likely associated with the presence of the pEh-Pc1-like plasmid, where this ARG was found to be conserved, and was exclusive to isolates recovered after 2018 (Fig. 8a). Likewise, ARG-acquired resistance against beta-lactams (*CTX-M-15* and *TEM-1*) as well as fosfomycin (*FosA3*) and macrolides (*mphA*) was only detected in the genomes of isolates recovered after 2018 and 2021, respectively. Further exploration of annotated plasmids found these ARGs to be located within pEh-Pc1-like and pEi-103–86.2 plasmids, respectively, which were also detected in the same period (Fig. 8a, b).

### Virulome

The occurrence and distribution of virulence factors identified through VFDB in *E. ictaluri* genomes are presented in Table S6. A total of 76 putative virulence genes were detected and classified into six categories including adherence (*n*=12; 16%), antimicrobial activity/competitive advantage (*n*=1; 1%), effector delivery system (*n*=42; 55%), immune modulation (*n*=3; 4%), motility (*n*=15; 20%) and regulation (*n*=3; 4%). Of these genes, 39 (51%) were conserved across *E. ictaluri* and included virulence factors related to antimicrobial activity/competitive advantage (*acrB*), immune modulation (*gndA*, *kdsA* and *ugD*), type VI secretion system (T6SS; *clpV*, *tss*-family) and regulation (*fur*, *rcsB and rpoS*). Moreover, exploration of annotated plasmids revealed additional T6SS genes (*VgrG* and *Pvc8*) to be conserved on the pEi-2083–2-like plasmid, carried by six (19%) isolates (Fig. 7c). Genes associated with a type III secretion system (T3SS), T4SS and type V secretion system (T5SS) were also detected in *E. ictaluri*. Genes associated with T3SS (*Esa-*, *Esc-*, *Esr-* and *sct*-families) were detected in 65% (*n*=20) of genomes, where they were located on the chromosome. Further, additional T3SS genes (*EscD* and *EseI*) were also detected on five (71%) pEI-S97-773-1-like plasmids (Fig. 7d). Likewise, the T4SS gene families *tra* and *trb* were frequently detected across three independent plasmid types, including pEI-2234–3-like (93%; *n*=13), pEh-Pc1-like (100%; *n*=10) and pEi-103–86.2 (Figs7b  8a, b). The T5SS-associated two-component *E. tarda* haemolysin system was highly prevalent in the *E. ictaluri* chromosome, with both *EthA* and *EthB* detected in 94% (*n*=29) of genomes screened. The genomes of Ei-64 and Ei-104, recovered in 2020 and 2021, respectively, both lacked the haemolysin secretion/activator gene *EthB*. No significant difference was observed between the number of virulence genes and *E. ictaluri* lineages Ei-P1 (average 61±3 genes) and Ei-P2 (average 57±4 genes) (Wilcoxon, *P*>0.05). However, the presence of *motA* was found to be significantly associated with lineage Ei-P2 (two-tailed Fisher’s exact test, *P*=0.05).

No significant change was detected in the number of virulence genes detected within *E. ictaluri* genomes between 2001 and 2021 (Spearman’s ρ, *P*>0.05) (Fig. 11c). Despite this, several virulence factors were lost or acquired in *E. ictaluri* genomes over time. This included motility protein A, encoded by *motA*, which was absent in the genomes of isolates recovered after 2003. Moreover, three isolates recovered after 2019 harboured several genes involved in the CS31A capsule-like antigen (*clpC*, *clpE*, *clpF*, *clpH* and *clpL*) and K88 fimbriae (*faeD* and *faeJ*), with the latter virulence factor located within a modified pEI-2234–3-like plasmid (Fig. 8b). No correlation was observed between the number of virulence genes and ARGs in respective genomes (Spearman’s ρ, *P*>0.05) (Fig. 11d).

## Discussion

*E. ictaluri* has received increased attention in recent years as it continues to challenge the striped catfish sector in Vietnam [9], with the incidence of disease outbreaks caused by *E. ictaluri* in non-catfish species also on the rise [13,14, 59]. Comparative genomics with this bacterium is therefore vital to better understand the evolutionary and population dynamics of the pathogen, as well as the genomic drivers behind AMR development and pathogenicity, which will ultimately help to inform better disease control strategies. The newly sequenced genomes significantly contribute to the growing *E. ictaluri* genome database, especially within the context of Vietnam, and allow us to explore the evolutionary history and population dynamics of Vietnamese * E. ictaluri* over a 20-year period.

In the present study, we assigned seven distinct lineages to global *E. ictaluri* populations based on phylogenetic analysis. Several lineages were found to be either country- or host-specific, supporting previous findings of distinct genotypes within global *E. ictaluri* populations [18]. Further, phylogenetic analysis revealed a clear distinction between the genotypes of American and Asian isolates, which has been reported previously [19,21]. The largest *E. ictaluri* lineage, Ei-P1, was overrepresented by isolates from striped catfish in Vietnam, although this lineage was also detected in isolates from the same fish species in Thailand as well as other fish species in China and Japan. These findings were supported by results from *in silico* MLST where ST 26 was the predominant ST in the region and suggests that closely related populations are now well established and widely distributed within SEA. Whilst the nature and mechanisms behind this transnational spread remain to be elucidated, these findings highlight the importance of active surveillance and strict biosecurity practices to better understand and minimize emerging *E. ictaluri* outbreaks across SEA.

Two distinct *E. ictaluri* lineages were found to have circulated the striped catfish sector within Vietnam in the two decades since the disease was first reported. Bayesian phylogenomic analysis revealed all isolates sequenced to have an MRCA that first emerged in the 1950s, around the same time that extensive pond farming of striped catfish was becoming popular in Vietnam [3]. Given that wild-caught seed, indigenous to the Mekong and Bassac rivers, was traditionally used to stock production systems [60], it is possible that the MRCA was already circulating within natural stock populations and thus introduced to production systems through wild seed stocks, where it became established due to high fish density and optimal growth conditions within the production systems. Likewise, given the broad natural host range of *E. ictaluri*, including other members of the *Pangasiidae* family [61], the MRCA may have originated in the other catfish species (e.g. *Pangasius bocourti* or *Pangasius conchophilus*) often farmed in polyculture with striped catfish during the early days of catfish farming in Vietnam [3]. The close phylogenetic relatedness of striped catfish to other *Pangasius* species means that they likely share similar traits including immune or antigenic mechanisms, which make them more susceptible to infection by the same pathogen [62].

In the present study, mutation rates for *E. ictaluri* were estimated at 1.5×10^−6^ substitutions per site per year. Whilst no previous estimates have been reported for *E. ictaluri* using similar methods, these estimates were higher when compared with other *Enterobacteriales* members, including *Klebsiella pneumoniae* (2.99×10^−7^) and *Salmonella enterica* (1.78×10^−7^ to 8.02×10^−8^) [63]. Despite these mutation rates, findings from this study show an ongoing decline in *E. ictaluri* population size within the striped catfish sector, concurrent with the predominance of a single dominant lineage in recent years. The production sector has witnessed little change in farming practices since the adoption of hatchery-produced seed, intensive pond culture techniques and high-quality feeds, between 2001 and 2005 [3,64, 65]. The timing of these events is in line with when unique SNPs were first detected in the *E. ictaluri* genome, as well as the period of more rapid decline in *E. ictaluri* population size, observed in this study. Our findings therefore suggest that whilst these husbandry changes were sufficient to intensify the production of striped catfish, the stability of the production sector, or lack of disruption through further changes in farming practices, over the last 20 years, has inadvertently created an ideal niche for *E. ictaluri* to persist with limited evolutionary pressures.

Whilst there was a high level of gene conservation within *E. ictaluri*, evident through high chromosome synteny and large core genome, the diverse mobilome detected may be a potential driver of genomic diversification in the pan-genome of emerging *E. ictaluri* populations within Vietnam. Indeed, nine plasmid types were detected across the genomes sequenced in this study, more than previously reported for this bacterial species [18,22]. Moreover, the presence of four plasmids from *E. ictaluri* associated with other hosts or countries demonstrates a wide dissemination of these plasmids within the global aquaculture sector, likely through the movement of seed stocks or live animals. The conservation of a pEI-2083–1-like plasmid within *E. ictaluri* populations circulating in Vietnam over 20 years suggests that this plasmid may play an important role in pathogenesis within the striped catfish host. Given the facultative intracellular nature of *E. ictaluri* in striped catfish [6], the presence of a NEL-type E3 ubiquitin ligase, which has remained well conserved in this plasmid, would support this theory as this enzyme family is known to disrupt eukaryotic cell signalling [66] and thus could allow the bacterium to evade host defences. The present study also provides an insight into the genomic drivers behind the divergence between American and Asian *E. ictaluri* populations, as the 5.6 kb plasmid associated with channel catfish isolates in the USA [21] was absent in Vietnamese *E. ictaluri* isolates recovered after 2005. These findings corroborate similar results of a recent study [22], which also found Vietnamese *E. ictaluri* isolates recovered after 2017 to lack a 5.6 kb plasmid and the associated virulence genes *EscD* and *EseI*. Given the fitness cost associated with plasmid maintenance [67], it is likely that this plasmid was lost in Vietnamese *E. ictaluri* populations over time and eventually replaced by a plasmid that provided a superior advantage to the fitness or pathogenicity of the host bacterium. Such plasmids could include the non-*E. ictaluri* plasmid types detected in isolates recovered after 2018, which carried a more diverse set of ARGs and virulence mechanisms. Whilst the presence of closely related *E. coli* plasmids in Ei-103 and Ei-104 supports previous findings in Vietnamese *E. ictaluri* recovered around a similar time [22], the detection of an *E. tarda-*like plasmid has not been reported previously. Although not identical to the pEh-Pc1 plasmid, the high sequence coverage of this plasmid with those found in *E. ictaluri* suggests a similar ancestry for both plasmid types and potential transmission between the two species in the past. This is highly probable given that both species have overlapping host and geographical ranges within the aquatic environment [68,69] and thus could have direct contact with each other.

The detection of ARGs against ten antibiotic classes in isolates recovered by 2021 suggests the presence of multidrug-resistant (MDR) populations within the Vietnamese aquaculture sector. These findings support that of previous phenotypic antimicrobial susceptibility and genotyping work in the same region [12,19, 22] and highlight the importance of minimizing antibiotic use within this aquaculture sector, to reduce the risk of antibiotic treatment failure caused by AMR. This is particularly critical as this study also demonstrated that *E. ictaluri* populations have already acquired genetic drivers of resistance to popular antibiotic choices for fish farmers in Vietnam. In the present study, the ARG families *dfrA*, *sul* and *tet* against trimethoprim, sulphonamides and tetracyclines, respectively, were highly prevalent across *E. ictaluri* genomes. Similar ARG families were also reported in populations circulating the same region in the latter time period of this study (2017–2021) [22] and are in line with the frequent use of these antibiotic families in the Vietnamese aquaculture sector [10,70]. Moreover, these ARG families have persisted in *E. ictaluri* populations over the last two decades, illustrating that long-term use of these antibiotics may be driving AMR development within the sector. Further evidence supporting this trend was also found in predicted fluoroquinolone resistance, as the ARG *Qnrs1* was acquired by *E. ictaluri* via two different plasmids after 2009. Enrofloxacin is a popular fluoroquinolone antibiotic used across the global aquaculture sector, including Vietnam, where enrofloxacin use in the striped catfish sector can be traced back to at least 2009 [15,71], in line with when *Qnrs1* was first detected. Moreover, despite banning the use of this antibiotic compound in Vietnam in 2012 [72], *Qnrs1* was still present in the genomes of isolates recovered as recently as 2021. Given that enrofloxacin use was still occurring in the Vietnamese aquaculture sector as late as 2018 [73], these findings highlight the importance of enforcing antibiotic regulations to reduce the persistence and dissemination of resistance mechanisms between *E. ictaluri* populations. In recent years, Vietnam has become a leader amongst other SEA countries in the country’s efforts to improve antimicrobial stewardship. The country has witnessed significant policy and regulation changes around antimicrobial use in human and animal health through their national action plans, developed in line with the policy frameworks of large multi-national agencies including the Food and Agriculture Organization of the United Nations, the World Health Organization and the World Organisation for Animal Health [74]. As part of these political developments, there has been a drive to promote the research and development of alternative treatment strategies, including vaccines, to replace antibiotics in aquaculture [75]. As we start to see a shift in antimicrobial use with increased use of vaccines or other innovative biological products, it will be important to continue active surveillance of *E. ictaluri* populations to monitor how these alternative husbandry practices drive changes in bacterial evolution, particularly within the context of AMR.

Findings from this study revealed that whilst *E. ictaluri* virulence has remained relatively stable over two decades, temporal changes were observed in several virulence factors during this period. The gene *motA*, which has been implicated in driving the bacterial flagellar motor and by extension bacterial motility [76], was lost in *E. ictaluri* recovered after 2003. As such, the loss of *motA* would support phenotypic observations of Vietnamese *E. ictaluri*, which, unlike American isolates, are reported as non-motile [8,77]. However, further exploration of genomes revealed that all isolates carried core flagellar biosynthesis genes, suggesting that they have retained the capability to build these appendages. Previous work in other Gram-negative bacteria has revealed the loss of *motA* whilst retaining a flagellar structure to be an adaptation by certain pathogens to confer phagocytic evasion [78]. Intracellular pathogens are known to use a variety of mechanisms to evade the immune response of their host, allowing them to invade and replicate within host cells [79]. Therefore, given the intracellular nature of *E. ictaluri* in striped catfish [6], similar adaptations could therefore have been made by *E. ictaluri* to modulate the host immune system and interfere with phagocytosis, thereby facilitating pathogenesis.

Comparative analysis of sequenced genomes also revealed that several isolates recovered in 2020 and 2021 had acquired, via a modified pEI-2234–3-like plasmid, genes, which encode for the outer membrane proteins (OMPs) required for the biosynthesis of a K88 fimbriae. In *E. coli*, the K88 (or F4) fimbriae is the most prevalent type of adhesin structure in enterotoxigenic *E. coli* (ETEC), where it allows for attachment to gut epithelial cells in mammalian hosts [80]. In fact, ETEC-associated diarrhoea is one of the leading disease challenges in swine and cattle production, causing significant economic losses [81]. The presence of such a virulence factor in *E. ictaluri* is therefore a concern for the striped catfish industry, as this fimbriae may result in hypervirulence in these isolates or changes in disease presentation, such as inducing enteritis through gut epithelium attachment and invasion, similar to other *Edwardsiella* species [82]. Whilst these changes would need to be validated *in vivo*, these findings highlight the need for further genomic surveillance of recent disease outbreaks to determine the prevalence of K88 fimbriae-related OMPs in *E. ictaluri*. If these OMPs are widely disseminated within *E. ictaluri* populations currently circulating in Vietnam, they may provide an opportunity for preventing and controlling the disease through the development of a vaccine to promote protection in striped catfish through mucosal immunity against *E. ictaluri*.

Despite the temporal changes in several virulence factors, immune modulation, effector delivery systems and regulation were found to be the major contributors to virulence in Vietnamese *E. ictaluri*. These were expected given the facultative intracellular nature of *E. ictaluri*, which invade and replicate within fish macrophages [6]. As such, the detected core genes involved in capsule formation, LPS, iron uptake and urease activity would be valuable for pathogenicity, supporting our existing understanding of this bacterium [69,83,85]. Amongst the suite of virulence genes detected, genomic elements of bacterial secretion systems, including T3SS and T6SS, were detected across the isolate collection, supporting previous findings [18,20,23]. Collectively, the protein secretion systems of Gram-negative bacteria play an essential role in communication, metabolism and pathogenesis [86]. Indeed, previous work on T3SS and T6SS in *E. ictaluri* has revealed these secretion systems to be vital to pathogenesis, functioning in cell attachment, invasion, stress resistance and survival [87,88].

Further comparison of genomes revealed the presence of several *tra*-family genes, associated with a T4SS [89], on three independent plasmid types carried by the *E. ictaluri* isolates recovered over two decades. The T4SS comprises a versatile set of cellular structures, which function in bacterial competition, horizontal gene transmission (HGT) and pathogenesis, by mediating the transfer of DNA and macromolecules to bacterial or eukaryotic cells [90]. The detection of *traD* was in stark contrast to previous work that did not detect *virD4* (a homologue of *traD*) in other *E. ictaluri* isolates from Vietnam [20,22], although this is likely due to isolates in these studies lacking the respective plasmids identified in this study. The gene *traD* encodes for an ATPase coupling protein, and its presence suggests that the T4SS detected in this study operates in a DNA transfer mode, as described for other species [91]. Given that the T4SS was located on plasmids which also carried a diverse array of ARGs, there is a risk that these ARGs could thus be widely disseminated within *E. ictaluri* populations circulating in Vietnam or be transmitted to other bacterial pathogens through HGT in the future, further driving AMR development within the striped catfish sector. Indeed, similar T4SSs have recently been associated with the transfer of multi-drug resistance in other *Edwardsiella* species [92]; thus, *E. ictaluri* could also act as an ARG reservoir and diver of ARG transmission. These findings therefore emphasize the importance of continued surveillance of *E. ictaluri* within the Vietnamese aquaculture sector, to monitor T4SS-driven AMR development.

The T5SS is one of the simplest but most abundant secretion systems in bacteria that can secrete a diverse array of substrates including toxins and thus contributes to the virulence of bacterial pathogens [93]. The presence of a highly conserved T5SS-associated *EthA-B* haemolysin system across the *E. ictaluri* genomes investigated was unexpected as these genes have not been detected in *E. ictaluri* previously. However, a similar two-component haemolysin system, encoded by *EihA* and *EihB*, has been detected in *E. ictaluri* isolates recovered from channel catfish in the USA [94]. Given the lack of *E. ictaluri* genes deposited within the VFDB and the high sequence homology between the two haemolysin systems (99% coverage and >88% identity), it is likely that these genes were mis-identified during the screening process. Indeed, further sequence alignment revealed the haemolysin system detected in this study to be identical to the system encoded by *EihA-EihB*. As these genes have not been reported previously in *E. ictaluri* from striped catfish or SEA [18,22], these findings therefore represent the first report of a T5SS-associated haemolysin system in Vietnamese *E. ictaluri*, although the function of this system in bacterial pathogenesis needs further validation. In other *Edwardsiella* species, similar haemolysin systems function in cell invasion and regulating T3SS and T6SS [95]; thus, similar functionality may also exist for *E. ictaluri*. Moreover, these findings also illustrate the need for a curated virulence gene database for *E. ictaluri* to allow for accurate and informative surveillance of this pathogen over time, a gap highlighted by others working in the same field [22].

In conclusion, the presence of MDR *E. ictaluri* populations, together with the emergence of new virulence mechanisms in recently circulating isolates, represents a significant threat to the production of striped catfish in Vietnam. Closely related *E. ictaluri* populations are widely dispersed through SEA with a clear phylogenetic distinction to those in USA, supporting our existing understanding of global *E. ictaluri* lineages. Moreover, genome-wide analysis of Vietnamese *E. ictaluri* isolates recovered over a 20-year period has facilitated a better understanding of the evolution of this pathogen within the striped catfish sector. Findings from this study have revealed a lack of evolutionary pressure within the production sector, leading to reduced genomic divergence of *E. ictaluri* populations. Despite this, the gene contents in plasmids contribute to the pathogen’s genomic diversity. The policy and legislation changes announced by the Vietnamese government are likely to see considerable change in aquaculture governance, husbandry and infrastructure. As such, continued genomic surveillance of *E. ictaluri* will be paramount to build on the work of this study, to better understand how these changes may drive genomic diversification of the pathogen in the future.

## supplementary material

10.1099/mgen.0.001368 Uncited Supplementary Material 1.

## Abbreviations

AG: An Giang
AMR: antimicrobial resistance
ANI: average nucleotide identity
BNP: Bacillary Necrosis of *Pangasius*
BV-BRC: Bacterial and Viral Bioinformatics Resource Center
CGE: Center for Genomic Epidemiology
CT: Can Tho
DT: Dong Thap
ETEC: enterotoxigenic *Escherichia coli*
HGT: horizontal gene transmission
HPD: highest probability density
MCMC: Markov chain Monte Carlo
MDR: multidrug-resistant
ML: maximum-likelihood
MRCA: most recent common ancestor
NCBI: National Center for Biotechnology Information
NS: Nested Sampling
OMPs: outer membrane proteins
SEA: Southeast Asia
ST: sequence type
TG: Tien Giang
T3SS: type III secretion system
T4SS: type IV secretion system
T5SS: type V secretion system
T6SS: type VI secretion system
VFDB: Virulence Factor Database
VL: Vinh Long
